# Tumor-associated macrophage subtypes on cancer immunity along with prognostic analysis and SPP1-mediated interactions between tumor cells and macrophages

**DOI:** 10.1371/journal.pgen.1011235

**Published:** 2024-04-22

**Authors:** Liu Xu, Yibing Chen, Lingling Liu, Xinyu Hu, Chengsi He, Yuan Zhou, Xinyi Ding, Minhua Luo, Jiajing Yan, Quentin Liu, Hongsheng Li, Dongming Lai, Zhengzhi Zou

**Affiliations:** 1 MOE Key Laboratory of Laser Life Science & Institute of Laser Life Science, College of Biophotonics, South China Normal University, Guangzhou, China; 2 Genetic and Prenatal Diagnosis Center, Department of Gynecology and Obstetrics, First Affiliated Hospital, Zhengzhou University, Zhengzhou, China; 3 Department of Hematology, The Third Affiliated Hospital of Sun Yat-sen University & Sun Yat-sen Institute of Hematology, Guangzhou, China; 4 Sun Yat-sen University Cancer Center, State Key Laboratory of Oncology in South China, Collaborative Innovation Center for Cancer Medicine, Guangzhou, China; 5 Department of Breast Surgery, Affiliated Cancer Hospital & Institute of Guangzhou Medical University, Guangzhou, China; 6 Shenshan Medical Center and Department of Gastrointestinal Surgery, Sun Yat-sen Memorial Hospital, Sun Yat-sen University, Guangzhou, China; 7 Guangdong Provincial Key Laboratory of Laser Life Science, College of Biophotonics, South China Normal University, Guangzhou, China; 8 Guangzhou Key Laboratory of Spectral Analysis and Functional Probes, College of Biophotonics, South China Normal University, Guangzhou, China; Brigham and Women’s Hospital Department of Medicine, UNITED STATES

## Abstract

Tumor-associated macrophages (TAM) subtypes have been shown to impact cancer prognosis and resistance to immunotherapy. However, there is still a lack of systematic investigation into their molecular characteristics and clinical relevance in different cancer types. Single-cell RNA sequencing data from three different tumor types were used to cluster and type macrophages. Functional analysis and communication of TAM subpopulations were performed by Gene Ontology-Biological Process and CellChat respectively. Differential expression of characteristic genes in subpopulations was calculated using zscore as well as edgeR and Wilcoxon rank sum tests, and subsequently gene enrichment analysis of characteristic genes and anti-PD-1 resistance was performed by the REACTOME database. We revealed the heterogeneity of TAM, and identified eleven subtypes and their impact on prognosis. These subtypes expressed different molecular functions respectively, such as being involved in T cell activation, apoptosis and differentiation, or regulating viral bioprocesses or responses to viruses. The SPP1 pathway was identified as a critical mediator of communication between TAM subpopulations, as well as between TAM and epithelial cells. Macrophages with high expression of SPP1 resulted in poorer survival. By *in vitro* study, we showed SPP1 mediated the interactions between TAM clusters and between TAM and tumor cells. SPP1 promoted the tumor-promoting ability of TAM, and increased PDL1 expression and stemness of tumor cells. Inhibition of SPP1 attenuated N-cadherin and β-catenin expression and the activation of AKT and STAT3 pathway in tumor cells. Additionally, we found that several subpopulations could decrease the sensitivity of anti-PD-1 therapy in melanoma. SPP1 signal was a critical pathway of communication between macrophage subtypes. Some specific macrophage subtypes were associated with immunotherapy resistance and prognosis in some cancer types.

## Author summary

Macrophages are natural immune cell-specialized, phagocytic cells, and many studies have been conducted to analyze the functional role of macrophages, but integrating macrophages from multiple cancers to analyze the molecular functions and interactions of macrophages is poorly known.We clustered and typed macrophages using single-cell RNA sequencing data from three different tumor types. Gene Ontology-Biological Processes and Cell Chat were used for functional analysis and communication of TAM subpopulations, respectively. We found heterogeneity of TAM and identified 11 subtypes and their impact on prognosis.The SPP1 pathway is recognized as a key mediator of communication between TAM subpopulations as well as between TAM and epithelial cells, and macrophages with high expression of SPP1 have a lower survival rate. Through in vitro studies, we also found that SPP1 mediated interactions between TAM clusters and between TAM and tumor cells. SPP1 promoted the tumor-promoting ability of TAM and increased PDL1 expression and stemness of tumor cells. In addition, we performed gene enrichment analysis using the REACTOME database and identified subpopulations that decrease the sensitivity of melanoma patients to anti-PD-1 therapy. Some specific macrophage subtypes are associated with immunotherapy resistance and prognosis in certain cancer types.

## Introduction

The tumor microenvironment (TME) consists of a diverse range of immune and non-immune stromal cells. TME can mediate immune escape of tumor cells, promote tumor stem cell formation and enhance tumor metastasis, thus promoting tumorigenesis and progression [1]. Tumor therapy strategies for targeting and regulating myeloid cells include manipulating the recruitment of myeloid subpopulations, stimulating the function of myeloid cells, and modulating the population response of myeloid cells [2]. Macrophages are widely exist in TME, whose metabolites affect various biological functions of TME, altering the immune microenvironment of tumors [3]. Interactions between tumor cells and macrophages can influence the behavior of tumor cells, with macrophages playing a dominant role in some tumor immune microenvironment beyond lymphocytes [4]. Therefore, an in-depth investigation of macrophages is important. Recent studies have shown that macrophages exhibit functional heterogeneity in different tissue environment, and this affects their ability to respond to metabolism [5–7], implying that the functions performed by macrophages in different TMEs may be different. Current studies have outlined the phenotypes and functions of macrophages and their impacts on cancer macrophages according to their different polarization states [8, 9]. Macrophages can be polarised into a pro-inflammatory M1 phenotype to combat pathogens. Besides, macrophages can also be polarised into an anti-inflammatory M2 phenotype to repair damaged tissue, thus acting as a tumor-supporting role [10, 11]. Macrophage polarization is regulated through complex interactions among various cytokines, chemokines and signaling molecules [12]. Macrophages are activated by IFN-γ, lipopolysaccharide and TNFα to promote the M1 phenotype, and with the activation of IL4 and IL13, macrophages at the site of injury show the M2 phenotype [13, 14]. Tumor-associated macrophages (TAMs) are macrophages recruited from circulating monocytes in tumors that are able to promote malignancy and tumor progression under the influence of cancer. In addition, TAMs are an important part of the immune cells in the TME of solid tumors, targeting TAMs can eliminate immunosuppressive factors in TME and stimulate anti-tumor effect of immune cells, enhancing the efficacy of immune checkpoint inhibitors [15]. The combination of TAM-targeted drugs with conventional therapeutics and other immunomodulators has proven to be a promising strategy [16]. Therefore, it is necessary to strengthen research on the pro-tumor mechanism of TAM, and make TAM targeted therapy an important supplement to traditional anticancer drugs [17]. Studies suggest that TAM is heterogeneous and functionally diverse. Therefore, to identify specific biomarkers for different subtypes of TAM is necessary for further research on macrophages in the future.

In recent years, the use of single-cell RNA sequencing (scRNA-seq) technology has allowed for the detection and analysis of various genetic components in organisms and the treatment of diseases. ScRNA-seq has become one of the most popular genomic tools for dissecting transcriptomic heterogeneity, with utility in dissecting intra-tumor heterogeneity and the tumor immune microenvironment at single-cell resolution in a wide range of tumors and the ability to identify rare cell types and cell states in tumors[18]. Furthermore, scRNA-seq data can eliminate technical noise to quantify intercellular heterogeneity of each gene [18–20]. Multiple studies have used scRNA-seq to analyze the functional heterogeneity of macrophages, glioma [21], liver cells [22], non-small cell lung cancer (NSCLC)[23], and HBV-associated human hepatocellular carcinoma [24]. Although TAM has been analyzed separately for each tumor, the similarities and differences in expression profiles and molecular functions of macrophage subsets in different cancer types remain to be determined. In addition, clinical outcomes of immunotherapy based on TAM subtypes still require further investigation.

In this study, we utilized a combination of single-cell tumor data from three different cancers to characterize the molecular and biological features of different macrophage subtypes in the TME. The molecular and biological features of different TAM subtypes were identified by distinguishing them into different subpopulations based on gene expression differences. Based on cellular communication analysis, we identified a possible role of the SPP1 pathway between macrophages and tumors. Using the TCGA database, we further characterized the prognostic value of signature genes in different subpopulations of TAM, providing new insights into how TAM can be targeted in specific cancer types. We found that TAM subtypes in specific cancer types can activate their specific functional pathways to regulate the TME and sensitivity to immunotherapy.

## Results

### General overview of the integration of macrophages from different cancers

**Fig 1:**
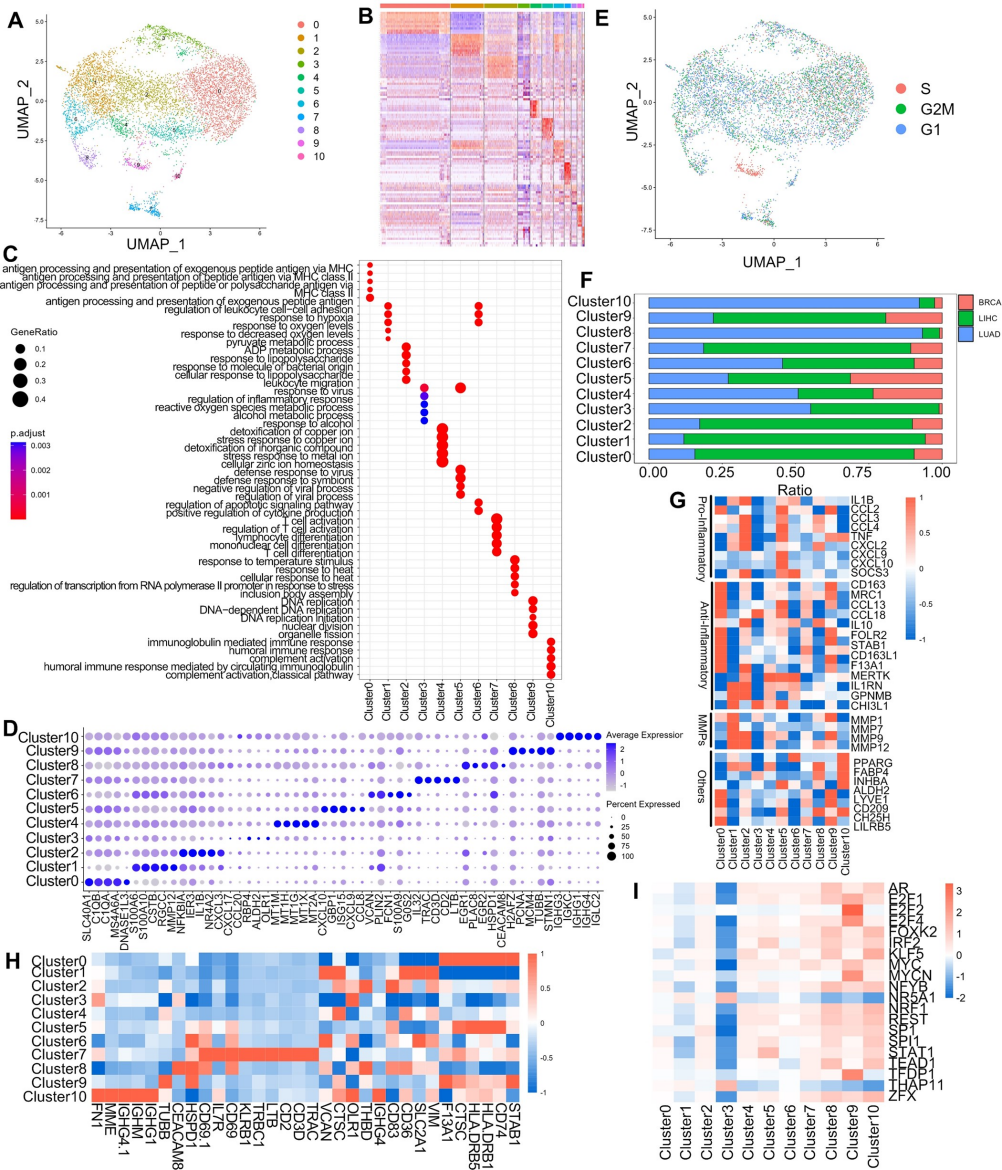
Tumor macrophage typing, marker genes and molecular functions. (A) UMAP shows the subpopulations of multiple tumor macrophages (breast cancer, liver cancer, and lung adenocarcinoma) after integration. (B) Heat map depicting the marker genes of different subpopulations. Different colors represent different Clusters, from Cluster0-Cluster10. (C) Dotplot depicting the functions of different subpopulations enriched for analysis. (D) Dotplot showing the top 5 marker genes of different subpopulations. (E) UMAP depicts the cell cycle of all macrophages. (F) Representation of different cancers in subgroups. (G) Heatmap of gene expression under specific functions. (H) Heatmap of genes encoding cell surface proteins. (I) Heatmap depicting the top 20 transcription factors that differ more significantly between different subpopulations.

To better understand the relationships and differences between macrophages in different cancers, we integrated multiple cancer datasets and screened for macrophage data while eliminating batch effects (S1 Fig). A total of 7801 macrophages from three cancer types (breast cancer, liver cancer, and lung adenocarcinoma) were integrated using Seurat, and 11 macrophage subtypes were identified as cluster 0–10 (Fig 1A and 1B). These macrophage subtypes were all present in the three cancer types with different abundance (Fig 1F and S1 Table), indicating the feasibility of our integration method. The largest proportion was Cluster0 (35%), which had a high expression of resident tissue macrophages markers (C1QA, C1QB) associated with T cell activation, inflammatory regulation, antigen processing and presentation (Fig 1C and 1D)[25]. Cluster1 (16.7%) had higher expression of TAMs markers (CSTB, RGCC) involved in lipopolysaccharide response as well as ADP metabolic processes (Fig 1C and 1D)[25]. Cluster2 (16.7%) expressed several markers of resident tissue macrophages (NR4A2, NR4A3, CXCL2, CXCL3, TNFAIP3, GPR183, CHMP1B) (Fig 1C and 1D), but it expressed fewer those marker genes compared to Cluster0, so they were analysed in subsequent functional analysis. Cluster0 expressed genes which were mainly enriched in cellular responses to stress and stimuli, while Cluster2 expressed genes related to the expression and modification of rRNA (Fig 1C)[25]. Cluster3 (6.0%) expressed TAMs marker gene OLR1 and was involved in viral responses as well as alcohol responses (Fig 1C and 1D) [25]. Cluster4 (5.7%) expressed most metallothioneins genes (MT1M, MT1H, etc.) and was involved in the action on metal ions (Fig 1C and 1D). Cluster5 (5.5%) expressed higher M1-type macrophages markers (CXCL9, CXCL10) for regulation of viral biological processes and responding to viruses (Fig 1C and 1D) [25]. Cluster6 (5.2%) was a group of monocyte-like macrophage expressing high levels of monocyte signature genes (VCAN, FCN1, S100A9) involved in response to oxygen levels and regulation of apoptotic signaling pathways and cytokines (Fig 1C and 1D)[25]. Cluster7 (3.2%) expressed high levels of cytotoxic genes (IL32) involved in T-cell activation and differentiation process (Fig 1C and 1D) [25]. Cluster8 (2.7%) expressed a high level of monocyte marker genes (HSPAIA) involved in oxygen and temperature regulation (Fig 1C and 1D)[25]. Cluster9 (2.3%) expressed a high level of proliferative genes (H2AFZ, PCNA, MCM4, MCM5, MCM56) associated with cell proliferation processes (Fig 1C) [25], and cell cycle analysis used scRNA-seq data also revealed that this subpopulation was enriched in S phase of cell cycle (Fig 1C and 1E). Cluster10 (1.0%) expressed a high level of selective immunoglobulin genes (IGHG3, IGHG1, IGHM, IGHG4, IGHD) involved in immune response and complement activation (Fig 1C and 1D)[25].

Our analysis focused on examining the gene expression profiles of two distinct subpopulations (Cluster0, Cluster2) across various cancer types. Surprisingly, even within the same category of tissue resident macrophages, we observed differential gene expression patterns among macrophages originating from distinct sources (S2A and S2B Fig). This discovery suggested notable variations in the gene expression profiles of analogous macrophages derived from different tumors. In our investigation, we examined the distribution of distinct tissue-resident macrophages across various tumor types. Specifically, we characterized tissue-specific macrophages present in breast cancer, denoted as FOLR2+ macrophages [26], hepatocellular carcinoma tissue-resident macrophages (Kupffer cells)[27], and tissue-resident macrophages within lung cancer recognized as alveolar tissue-resident macrophages [28]. Our analysis revealed a higher expression of tissue-resident macrophages in both Cluster0 and Cluster2 subpopulations. Notably, these different tumor-specific macrophages exhibited similar expression patterns across both subpopulations (S2C Fig). We performed differential analysis and KEGG enrichment analysis on several clusters exhibiting similar expression patterns. Specifically, differential analysis was conducted on the following pairs of clusters: Cluster0 versus Cluster2, Cluster1 versus Cluster3, and Cluster6 versus Cluster8. This allowed the identification of distinct sets of differential genes within these clusters. Subsequently, we subjected these differential genes to enrichment analysis, unveiling divergent pathway enrichments between Cluster0 and Cluster2. Cluster0 showed enrichment in pathways associated with bacterial and viral infections, including systemic lupus erythematosus, staphylococcal infections, and antigen processing and presentation. On the other hand, Cluster2 was demonstrated to be involved in pathways related to the NF-κB pathway, the IL17 pathway, and infections linked to Kaposi sarcoma-associated herpesvirus. Both clusters appeared to be associated with distinct bacterial infections, showing divergent enrichment patterns. Relative to Cluster1, Cluster3 displayed pathways involved in bacterial and viral infections, as well as antigen processing and presentation. Conversely, Cluster6 was notably enriched in metabolic pathways alongside bacterial and viral infection-related pathways (S3 Fig).

We conducted a comprehensive analysis of macrophage subpopulations by annotating and functionally characterizing each subpopulation using a gene set associated with key macrophage-related biological processes reported in previous studies [29]. Our results revealed distinctive patterns of gene expression among the different clusters. The analysis revealed that Cluster0 macrophages exhibited higher expression levels of resident macrophages marker genes compared to other subpopulations, and expressed both high anti-inflammatory and partially pro-inflammatory genes. Furthermore, Cluster0 expressed the immunomodulators (CD209, CH25H, LILRB5), which were associated with innate and adaptive immunity, indicating their functional role in immune modulation (Fig 1G). Cluster1 expressed high levels of anti-inflammatory and pro-inflammatory genes, along with a unique subpopulation of MMP (Fig 1G). Cluster3 expressed high levels of TGF β coactivator INHBA, while Cluster4 expressed high levels of anti-inflammatory genes (Fig 1G). Cluster5 exhibited high levels of both anti-inflammatory and pro-inflammatory genes and immunomodulators (CD209, CH25H, LILRB5) (Fig 1G). Cluster6 expressed partial anti-inflammatory genes, and high expression of PPARG, which is an intrinsic alveolar macrophage marker (Fig 1G). Upon analyzing the data, we noticed that Cluster6 harboured a substantial count of LUAD macrophages, accompanied by a considerable presence in liver hepatocellular carcinoma (LIHC) and a lower proportion in breast cancer (BRCA) samples (Fig 1F). Additionally, our observations revealed elevated expression levels of the monocyte signature genes S100A9 and FCN1 within Cluster6 compared to other subpopulations (Fig 1D). Consequently, we suggested that Cluster6 was not just alveolar resident macrophages but might be generalized monocyte-like macrophages. Cluster7 exhibited high expression of anti-inflammatory genes, while Cluster8 expressed primarily pro-inflammatory genes (Fig 1G). Cluster9 showed high expression of anti-inflammatory genes and MMP genes, along with high expression of the immunomodulator (CD209, CH25H, LILRB5), involved in innate and acquired immunity (Fig 1G). Finally, Cluster10 exhibited high expression of the intrinsic alveolar macrophage marker PPARG, anti-inflammatory genes (FABP4, ALDH2, TGF β) coactivator INHBA, and immunomodulator CH25H (Fig 1G). Furthermore, the predominant subset of Cluster10 macrophages originated from LUAD samples, as depicted in Fig 1F and S1 Table. Our supposition was centered on the potential association between the macrophages derived from LUAD within Cluster10 and alveolar macrophages. These results indicated similarities and differences between different clusters, and each macrophage isoform expressed different levels of pro- or oncogenes (Fig 1G).

In addition to the aforementioned analyses, we utilized the Cell Surface Protein Atlas database to identify proteins produced by surface-tagged genes, providing a useful framework for future experiments such as identifying and isolating specific macrophage subtypes (Fig 1H). Moreover, we employed a gene set containing transcription factors (TFs) interacting with their targets and then infer TFs activity from gene expression data. Our findings revealed that among the 20 TFs with the greatest intercellular population variation, NR5A1 and THAP11 were highly expressed only in Cluster3, TFDP1 and E2F2 were highly expressed in Cluster9. We found the low expression of these transcription factors in Cluster3. Other transcription factors in Cluster3 also exhibited similar low expression (Fig 1I).

The above results demonstrated the transcriptomic and molecular characterization of multiple macrophage subtypes in different cancer types. Further, it was useful to separate different subpopulations of macrophages based on the difference of highly expressed genes between subpopulations.

### Cellular interactions of different clusters

**Fig 2:**
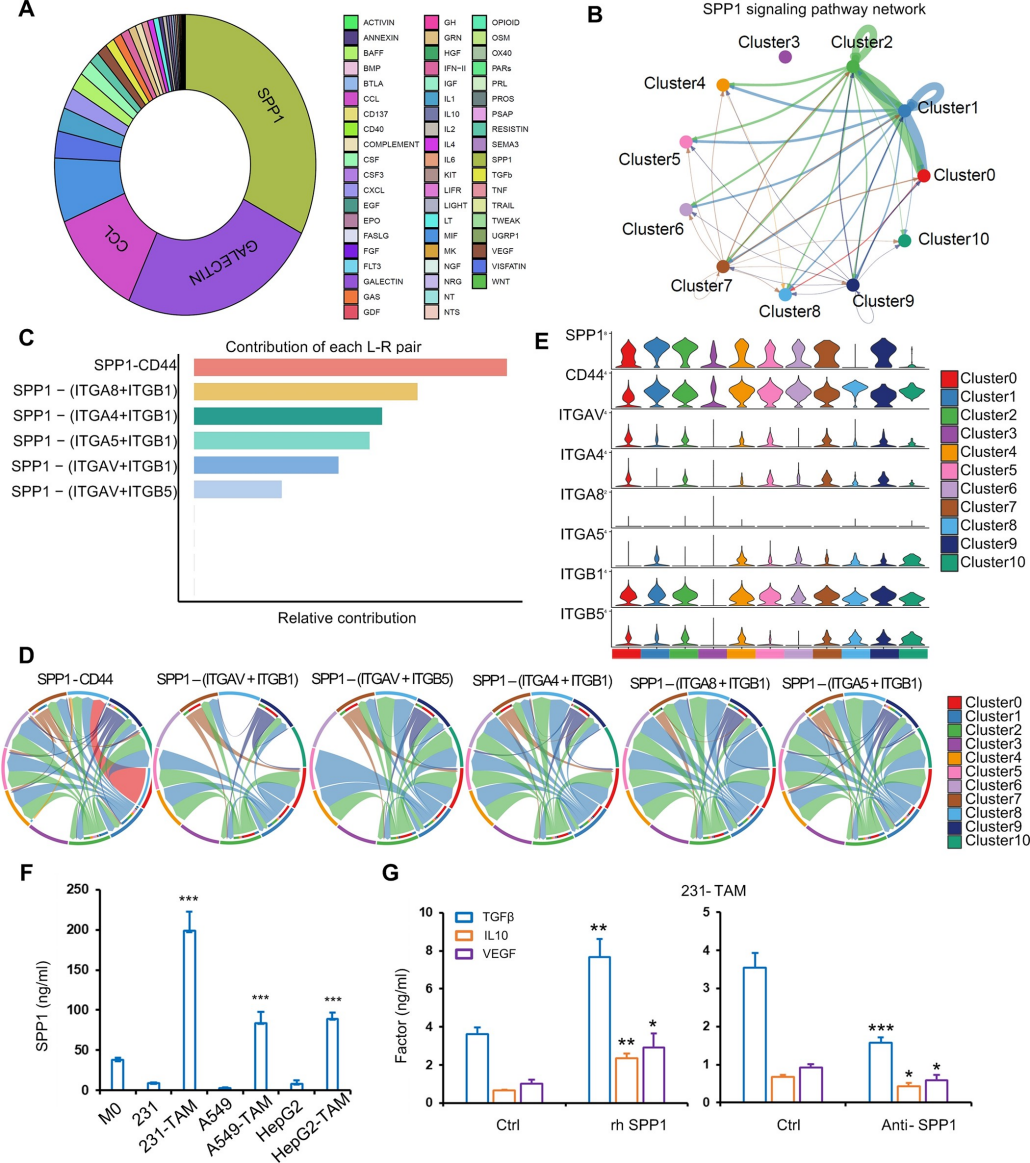
The strongest communication between different subgroups is through the SPP1 pathway. (A) Pie chart showing the percentage distribution of different signaling pathways. (B) Line graph showing the intercellular action of the SPP1 pathway. (C) Contribution of different receptors of SPP1 action. (D) SPP1 action with receptor CD44, ITGA8 and ITGB1, ITGA4 and ITGB1, ITGA5 and ITGB1, ITGAV and ITGB5. (E) Expression of different receptors in different subpopulations. (F) Human SPP1 levels in M0, tumor cells and TAM were detected by ELISA. (G) Human TGF β, IL10 and VEGF levels in MDA-MB 231 (231)-induced TAM treated by rh SPP1 protein and anti-SPP1 antibody were detected by ELISA. Data were shown as mean ± SD and are representative of three independent experiments. P values were calculated using the 2-tailed 2-sample t test. *P < 0.05, **P < 0.01, ***P < 0.001.

To investigate the cell-cell communications between macrophages in the TME, we utilized the CellChat analysis. The communication between different subpopulations of macrophages was assessed by examining the roles of receptors and ligands in cell-to-cell communication (S4A and S4B Fig). We observed the number and strength of communication between different subpopulations, which we classified into three outgoing patterns and two incoming patterns (S4C and S4D Fig). We analyzed the subpopulations of macrophages as ligands or receptors separately to determine their action types. We identified up to three outgoing patterns, with Cluster0, Cluster3, and Cluster7 classified as pattern 1 type, Cluster4, Cluster6, Cluster8, and Cluster10 as pattern 2 type, and Cluster1, Cluster2, and Cluster9 as pattern 3 type. Most of the pathways were more active in the subgroup of pattern 1, indicating that cells of Cluster0, Cluster3, and Cluster7 are more active as ligands for communication with other cells. We observed two incoming patterns: Cluster0, Cluster1, Cluster2, Cluster3, Cluster7, and Cluster9 were classified as pattern 1, while Cluster4, Cluster6, Cluster8, and Cluster10 were classified as pattern 2. The OX40, IL2, ACTICIN, GRN, ANNEXIN, and UGRP1 pathways were active for cell communication in cell populations with pattern 2 as incoming patterns (S4C and S4D Fig). To obtain more critical cell-cell interactions in TAM, we analyzed some signaling pathways and their receptor-ligand pairs to explore potential interactions between macrophages. We calculated the weight of each signaling pathway to the overall cellular interactions. Results showed that SPP1 signal pathway showed the maximum proportion of weight in all signaling pathways involved in TAM cluster interactions (Fig 2A and S2 Table). The connection between TAMs was complex, our findings provided valuable insights into the cellular interactions between different subpopulations of macrophages, which could contribute to a better understanding of the immune response.

### SPP1-mediated interaction between macrophage clusters

Since SPP1+ macrophages were shown to be important for the TME in previous studies [30], we further analyzed the SPP1 pathway in macrophages and analyzed SPP1+ macrophages in pan-cancer. We found that SPP1-CD44 made a major contribution to the SPP1 signaling pathway (Fig 2B–2D). Next, we analyzed the interactions between these ligand-receptor pairs among the 11 macrophage subtypes and found that SPP1 interacts with most of the receptors, except in Cluster3. In addition, we used a violin plot to visualize the signal gene expression distribution in the SPP1 signal pathway, which was inferred by CellChat. The results showed that SPP1 was expressed in most macrophages (Fig 2E). Moreover, we detected SPP1 expression in M0 macrophages, tumor cells (breast, lung and liver cancer) and TAM by ELISA *in vitro*. TAMs exhibited high levels of SPP1 relative to M0 macrophages and tumor cells (Fig 2F). To validate SPP1 mediated interaction between TAMs, TAMs induced by MDA-MB 231 cells were treated with recombinant human SPP1 (rh SPP1) protein. Tumor-promoting and anti-inflammatory factors TGF β, IL10 and VEGF were significantly stimulated by rh SPP1 protein but inhibited by anti-SPP1 antibody (Fig 2G). These results suggested that SPP1 mediated interaction between macrophage clusters.

### SPP1-mediated communication between TAM and tumor cells

**Fig 3:**
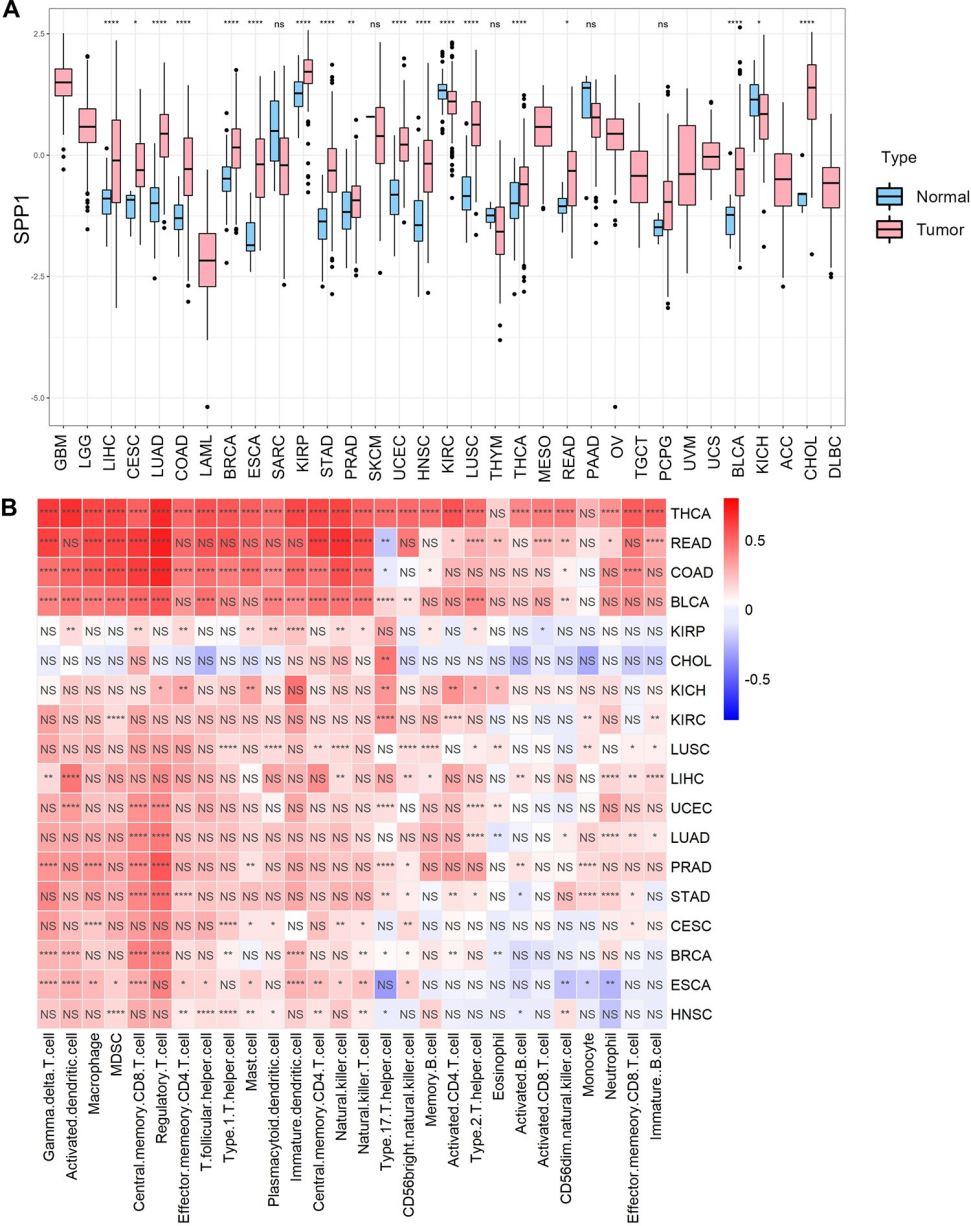
High SPP1 expression in some tumors may be associated with immune infiltration. A) Differential expression of SPP1 in tumors and normal in different cancers. (B) Correlation between SPP1 expression and immune infiltration. *P* < 0.05, *P* <0.01, *P* < 0.001, and *P* < 0.0001 were considered statistically significant (*, **, ***, ****).

To explore the expression of SPP1 in tumor tissues, we performed differential analysis of SPP1 in tumors and normal tissues and found significant differences in 18 cancer types in all TCGA data (Fig 3A). We then performed immune infiltration analysis on these 18 cancers and calculated the correlation between SPP1 expression and immune cell infiltration, and we found that SPP1 was positively correlated with immune cell infiltration including macrophage in most of these cancers (Fig 3B). Our above results indicated that TAM exerted higher levels of SPP1 expression than tumor cells. Macrophages accounted for a large proportion in solid tumor tissue, indicating a possible positive correlation between the expression of SPP1 and the infiltration of macrophages in solid tumors.

**Fig 4:**
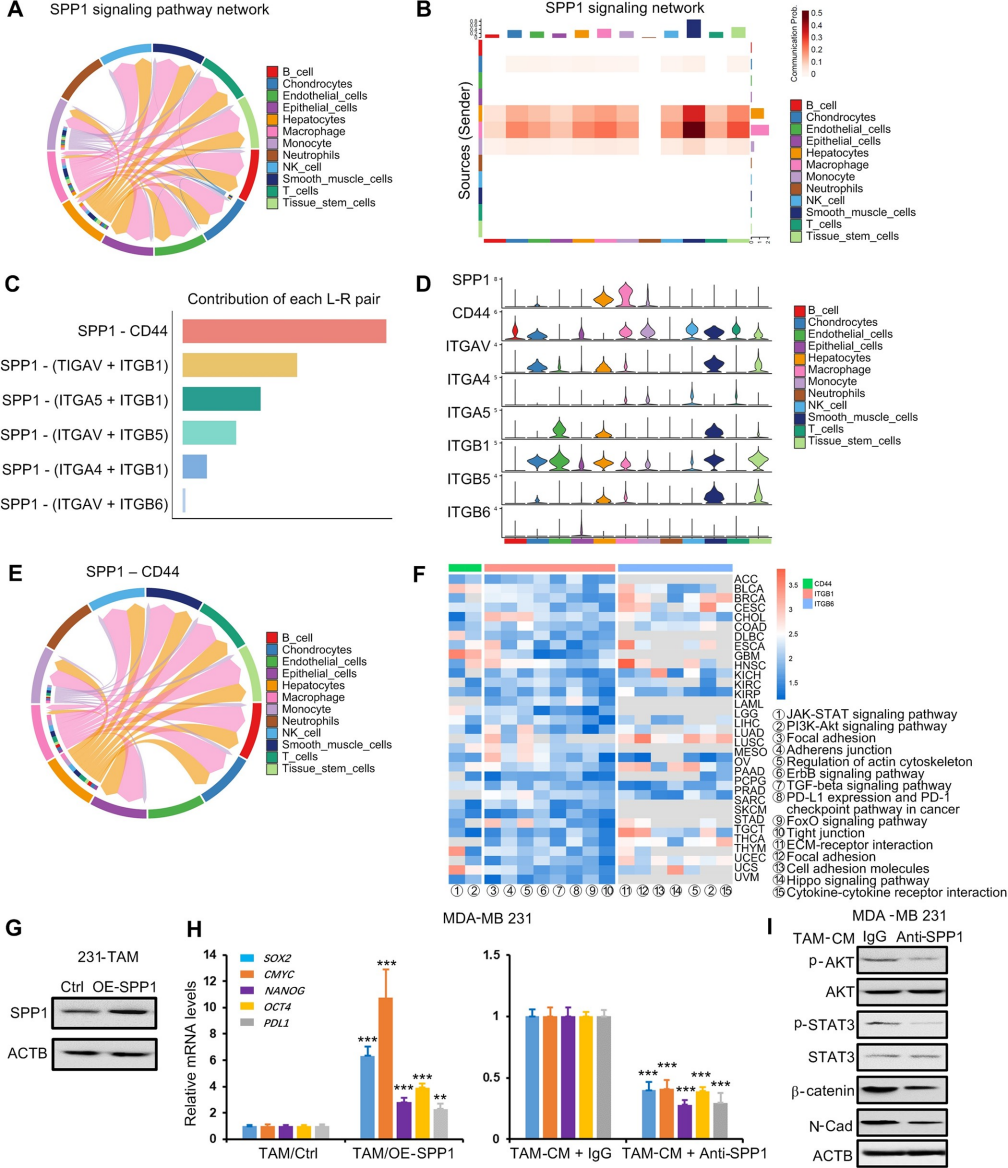
Intercellular communication between macrophages and epithelial cells via the SPP1 signaling pathway. (A-B) Chord diagrams and bar graphs showing the interaction scores of various cell types in cancer predicted by the SPP1 signaling pathway. (C) Contribution of different receptor-ligand pairs of the SPP1 signaling pathway. (D) Expression of receptor-ligands in the SPP1 signaling pathway in different cell types. (E) Chordal graph showing interaction scores in different cell types via SPP1-CD44 receptor-ligand pairs. (F) Normalized enrichment score (NES) of different pathways in various cancers. (G) SPP1 levels in MDA-MB 231 (231)-induced TAM with SPP1 overexpression were detected by Western blot. (H) 231 cells were treated for 48 h with condition media from 231-induced TAM with SPP1 overexpression, or condition media (with SPP1 antibody or without) from 231-induced TAM. SOX2, CMYC, NANOG, OCT4 and PDL1 were detected by RT-qPCR. (I) 231 cells were treated for 48 h with condition media (with SPP1 antibody or without) from 231-induced TAM. The indicated proteins were detected by Western blot. Data were shown as mean ± SD and are representative of three independent experiments. P values were calculated using the 2-tailed 2-sample t test. *P < 0.05, **P < 0.01, ***P < 0.001.

We chose to analyze single-cell sequencing data from three tumors where SPP1 expression was positively correlated with immune infiltration including Thyroid carcinoma (THCA), Colon adenocarcinoma (COAD), and prostate cancer (PRAD), respectively. Results revealed that SPP1 was mostly expressed in macrophages and monocytes, and less in epithelial cells (S5 Fig). This provided a possibility that macrophages-derived SPP1 made a greater contribution to SPP1 expression in the TME. In order to investigate the possible alteration of tumor status caused by TAM in the TME, we used the "CellChat" package to score the regulatory relationships between different cell types by known receptor-ligand pairs. Among the ligand-receptor pairs related to epithelial cells, TAM was the primary cell type for epithelial cell communication, and SPP1 was the most important ligand for their communication (Fig 4A and 4B and S3 Table). SPP1 binded strongly to CD44 and the integrin family (encoded by ITGAV/ITGB1, ITGAV/ITGB5, ITGAV/ITGB6, and ITGA5/ITGB1) and the predominance of SPP1-CD44 (Fig 4C–4E). We also analyzed the effect of SPP1 on prognosis in 33 cancers and found that high expression of SPP1 was positively associated with poor prognosis in most cancers (S6 Fig), suggesting that cell communication of TAM with tumor epithelial cells via SPP1 may lead to tumor progression. Finally, we performed KEGG enrichment analysis of SPP1 signaling pathway receptors (CD44, ITGB1, ITGB6) that were expressed in epithelial cells in 33 cancers, and extracted the intersections of the enriched pathways (S7 and S8 Figs). In all enriched pathways, 17 cancer-related pathways were identified (Fig 4F). Therefore, this raised a possibility that TAM-secreted SPP1 activated these pathways such as the JAK-STAT and PI3K-Akt signaling pathways, reduced focal adhesion protein and induced PDL1 expression in epithelial cells to contribute to tumor prognosis.

To further demonstrate that TAM communicated with tumor cells via SPP1, SPP1 was overexpressed in TAM cells (Fig 4G). Breast cancer MDA-MB 231 cells were treated with conditioned medium from SPP1-overexpressed TAM, and tumor stem cell markers (*SOX2*, *CMYC*, *NANOG*, *OCT4* gene) and *PDL1* genes were detected by RT-qPCR. Results showed that SPP1-overexpressed TAM obviously induced the five genes expression, whereas blocking SPP1 using antibody significantly decreased the gene expression induced by TAM (Fig 4H). In addition, we also showed that SPP1 antibody inhibited TAM-activated AKT and STAT3 pathways and TAM-induced N-cadherin and β-catenin expression in MDA-MB 231 cells (Fig 4I). These results suggested that SPP1 mediated communication between TAM and tumor cells.

### Prognostic association of different macrophage types in pan-cancer

**Fig 5:**
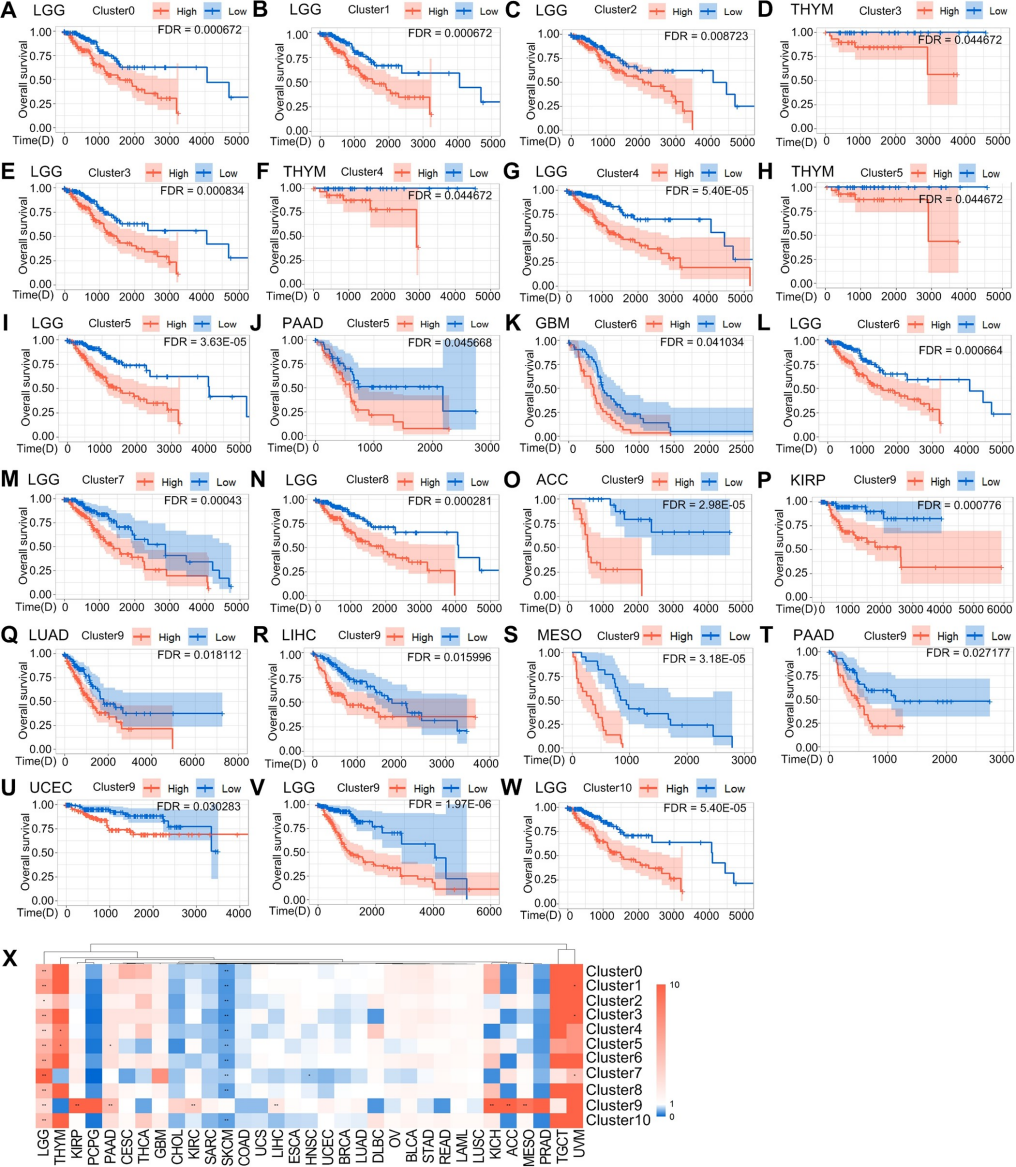
Specific Macrophage subtypes linked to clinical outcome in distinct cancer types. (A-W) Kaplan-Meier (KM) curves depicting differences in survival of highly and poorly expressed signature genes in multiple subpopulations in different tumors. The light-colored background area is the confidence interval of the probability of survival at each time point calculated by the KM method, which is the 95% confidence interval. (X) Clustering of tumor types by risk ratios of 11 macrophage subpopulations, Time(D) = Time (Days), colors indicate risk ratios (red = risk ratio >1 [poor prognosis], blue = risk ratio < 0.05, * FDR < 0.05, ** FDR < 0.01.

We investigated the cellular interactions and prognostic association of macrophage subtypes in 33 cancer types by grouping them based on their signature genes expression. As macrophages have both pro- and anti-cancer effects, we analyzed their expression levels to determine their association with tumor progression and clinical outcomes. Our analysis revealed that the signature genes expression varied among macrophage subtypes and cancer types. For instance, we found that the signature genes of Cluster0 had the highest expression in kidney clear cell carcinoma (KIRC) and the lowest expression in ocular melanomas (UVM)(S9A Fig), and high expression in lower grade glioma (LGG) was associated with poor prognosis (Fig 5A). Similarly, the signature genes of Cluster1 had the highest expression in KIRC and the lowest expression in UVM (S9B Fig), and high expression in LGG was also associated with poor prognosis (Fig 5B). The signature genes of Cluster2 had highest expression in KIRC, the lowest in UVM (S9C Fig), and high expression in LGG was associated with poor prognosis (Fig 5C). The signature genes of Cluster3 had the most highly expressed in LUAD, the least expressed in PRAD (S10A Fig), and highly expressed in thymoma (THYM) and LGG was associated with poor prognosis (Fig 5D–5E). In addition, we found that the signature genes of Cluster4 were the most highly expressed in KIRC, the least expressed in UVM (S10B Fig), and high expression in THYM, LGG was associated with poor prognosis (Fig 5F–5G). The Cluster5 signature genes was the most highly expressed in large B-cell lymphoma (DLBC) and least expressed in LGG and UVM (S10C Fig), and high expression in THYM, pancreatic cancer (PAAD), and LGG was associated with poor prognosis (Fig 5H–5J). Cluster6 had the highest expression in KIRC and the lowest expression in UVM and PRAD (S11A Fig), and high expression in glioblastoma (GBM), LGG led to poor prognosis (Fig 5K–5L). Cluster7 had the highest expression in DLBC and the lowest expression in UVM (S11B Fig), and high expression in LGG was associated with poor prognosis (Fig 5M). Cluster8 had the highest expression in DLBC and the lowest expression in PRAD and pheochromocytoma & paraganglioma (PCPG) (S11C Fig), and high expression in LGG was associated with poor prognosis (Fig 5N), Cluster9 had the highest expression in acute myeloid leukemia (LAML) and the lowest expression in PCPG and head and neck cancer (KICH)(S12B Fig), and high expression in adrenocortical cancer (ACC) and kidney papillary cell carcinoma (KIRP), LUAD, LIHC, mesothelioma (MESO), endometrioid cancer (UCEC) and LGG correlates with poor survival(Fig 5O–5V). The signature genes of Cluster10 were highest in BLDC and KIRC and lowest in UVM and LGG (S12C Fig), and high expression in LGG was associated with poor prognosis (Fig 5W). We observed that a few cancers, such as BRCA and UCEC, with high expression of Cluster7 signature genes were associated with better prognosis (S13A–S13B Fig), while high expression of Cluster9 signature genes in THYM was associated with better prognosis (S13C Fig). In contrast, high expression of the signature genes of all melanoma (SKCM) subpopulations, except for the signature genes of Cluster9, led to better prognostic outcomes (S13D–S13M Fig). High expression genes for different macrophage subgroups were found to be correlated with better prognosis in various cancer types, including BRCA, THYM, UCEC, and SKCM (S13 Fig). To further investigate these prognostic associations, we performed multivariate Cox proportional hazard modeling, adjusting for age and gender. Our results indicated that high expression of these signature genes was significantly associated with worse clinical outcomes in the aforementioned cancer types (S4 Table). Overall, our study provided valuable insights into the prognostic associations of different macrophage subtypes in pan-cancer. Further research is needed to understand the cellular interactions between these subtypes in different clusters and their potential implications for cancer treatment.

To improve the assessment of the prognostic association of the signature genes, we conducted univariate risk analysis on the gene signature of macrophage subpopulations. Our analysis revealed a high risk for all subpopulations in LGG while most macrophage subpopulations in SKCM showed a low risk (Fig 5X). These findings delineated the TAM landscape in various cancer types, revealing the dichotomous impacts of macrophage subtypes, which could either promoted or suppressed specific cancer types. This complexity underscored the multifaceted role of macrophages within the context of cancer. Consequently, there was a pressing need for targeted investigations tailored to individual cancer types to elucidate the precise role of macrophages. Such focused studies holded the potential to guide subsequent prognostic inquiries aimed at understanding the prognostic implications of macrophages in cancer.

### Molecular functions of different macrophage clusters in various tumors

**Fig 6:**
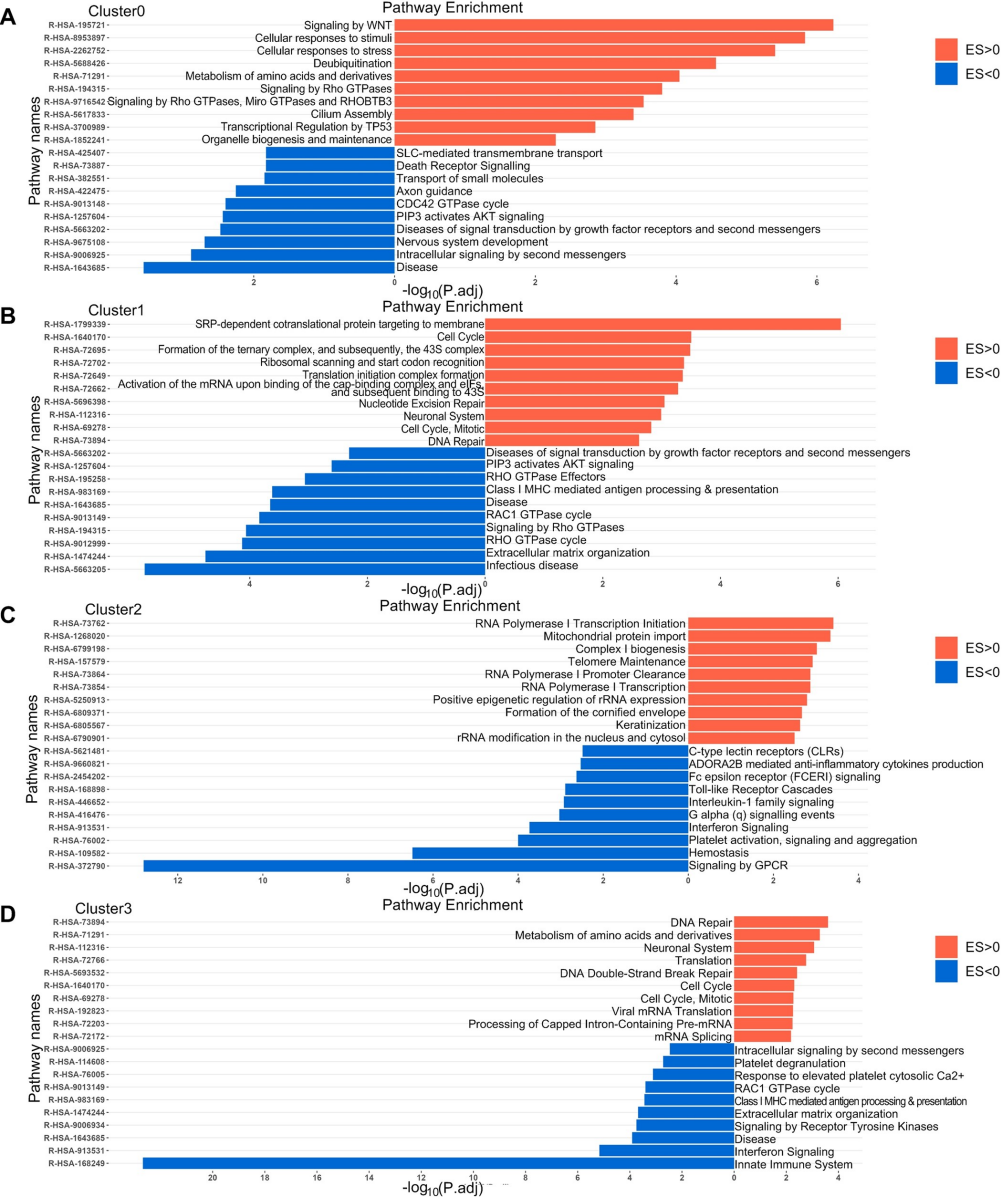
Networks of REACTOME terms enriched or depleted in tumors with high expression of different macrophage subtypes (Cluster0-Cluster3) signature genes. (A) Cluster0 results from SKCM and LGG. (B) Cluster1 results from SKCM and LGG. (C) Cluster2 results from SKCM. (D) THYM, SKCM and LGG results for Cluster3.

In our study, we aimed to investigate the impacts of macrophage subtypes on tumor prognosis and uncover underlying molecular mechanisms. To achieve this, we conducted enrichment analysis on cancer types that exhibited significantly poor or favorable prognoses. Our investigations unveiled distinct functional enrichments within each cluster, shedding light on their pivotal roles in various cellular activities and biological mechanisms. Cluster0 exhibited functional enrichment indicative of its involvement in intricate intracellular signaling networks, pivotal regulatory mechanisms governing cellular function, metabolic regulation, and responses to external stimuli (Fig 6A). Cluster1 emerged as a central player in diverse cellular processes encompassing protein synthesis, cell cycle regulation, DNA repair, and the development and function of the nervous system (Fig 6B). The functional enrichments observed in Cluster2 suggested its significance in a wide array of biological processes, including gene transcription and regulation, organelle function, and cytoskeletal reorganization (Fig 6C). In Cluster3, enriched functions pointed to its importance in pivotal biological processes such as DNA repair, cell cycle regulation, and RNA processing (Fig 6D). Cluster4 appeared to be significantly involved in RNA metabolism, processing and potentially viral infection, along with cell cycle regulation (S14A Fig). The functional enrichment of Cluster5 highlighted its critical role in sustaining mitochondrial biosynthesis, protein synthesis, and overall cellular activity (S14B Fig). Notably, enrichment in Cluster6 suggested its importance in nervous system development and signaling (S14C Fig). Likewise, the enriched characterization of Cluster7 implied its involvement in vital cellular processes like RNA processing, transcription, translation, and cellular metabolism (S14D Fig). The functional characterization of Cluster8 indicated its involvement in diverse biological processes encompassing cell signaling, stress responses, and regulation of nuclear receptors (S15A Fig). Cluster9’s functional characterization suggested its involvement in key biological processes such as cell metabolism, regulation of translation, and remodeling of the extracellular matrix (S15B Fig). Finally, the functional characterization of Cluster10 suggested its potential role in biological processes including nervous system development and regulation of the cell cycle (S15C Fig). The results of our analyses suggested that these macrophage subtypes exhibited significant differences in their impact on tumor progression, reflecting the differences between macrophage subtypes as well as the heterogeneity of macrophages among different tumors. Overall, our study provided valuable insights into the cellular interactions of different macrophage subtypes and their impacts on tumor development. These findings might have significant implications for cancer treatment and management.

### Associations of macrophage subgroups with immunotherapy sensitivity in different types of cancer

**Fig 7:**
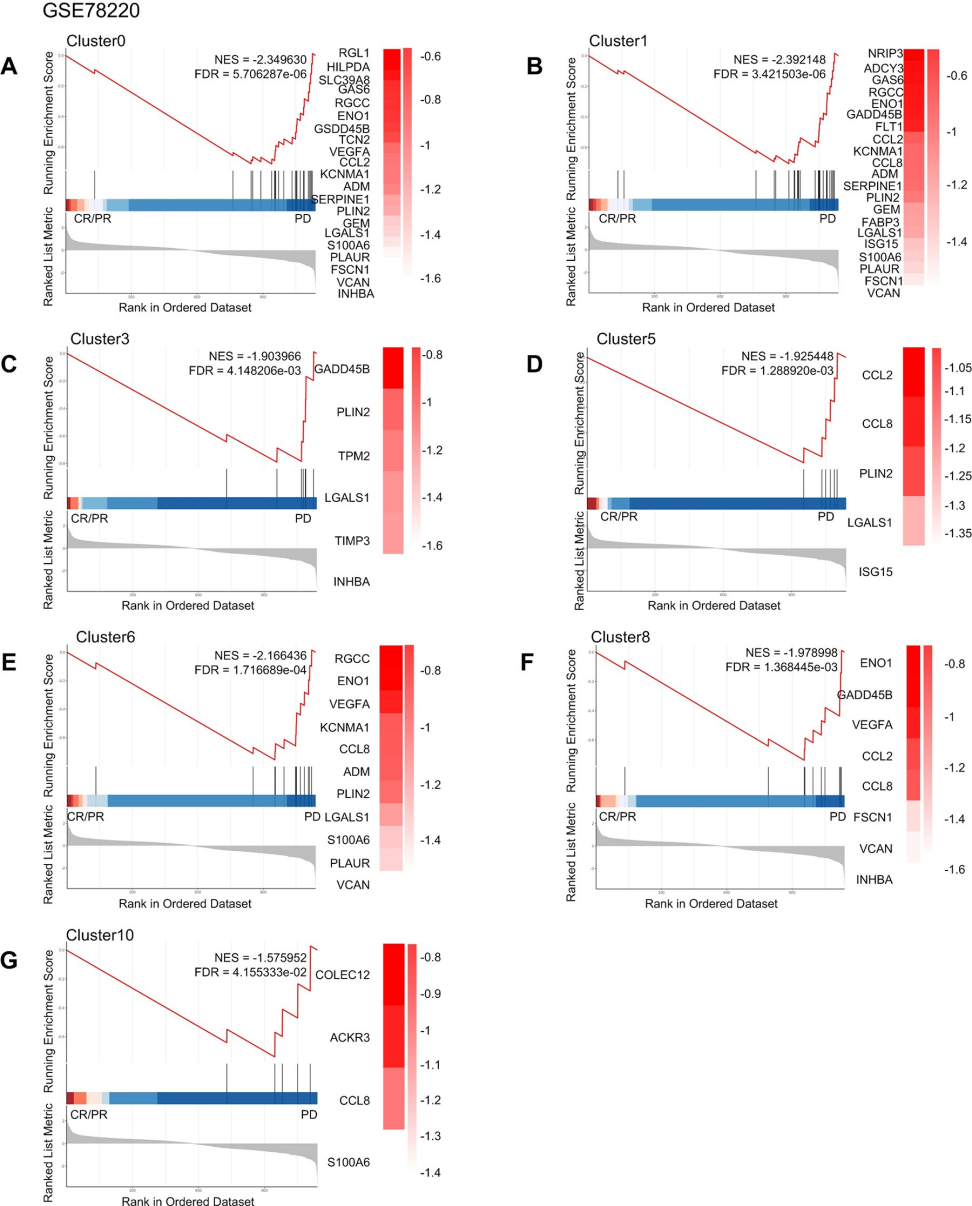
Macrophage subtypes of anti-PD-1 resistance in melanoma. Gene set enrichment analysis of anti-PD 1 treatment for melanoma showed that patients in complete remission (CR) and partial remission (PR) compared with those in patients with progressive disease (PD).

**Fig 8:**
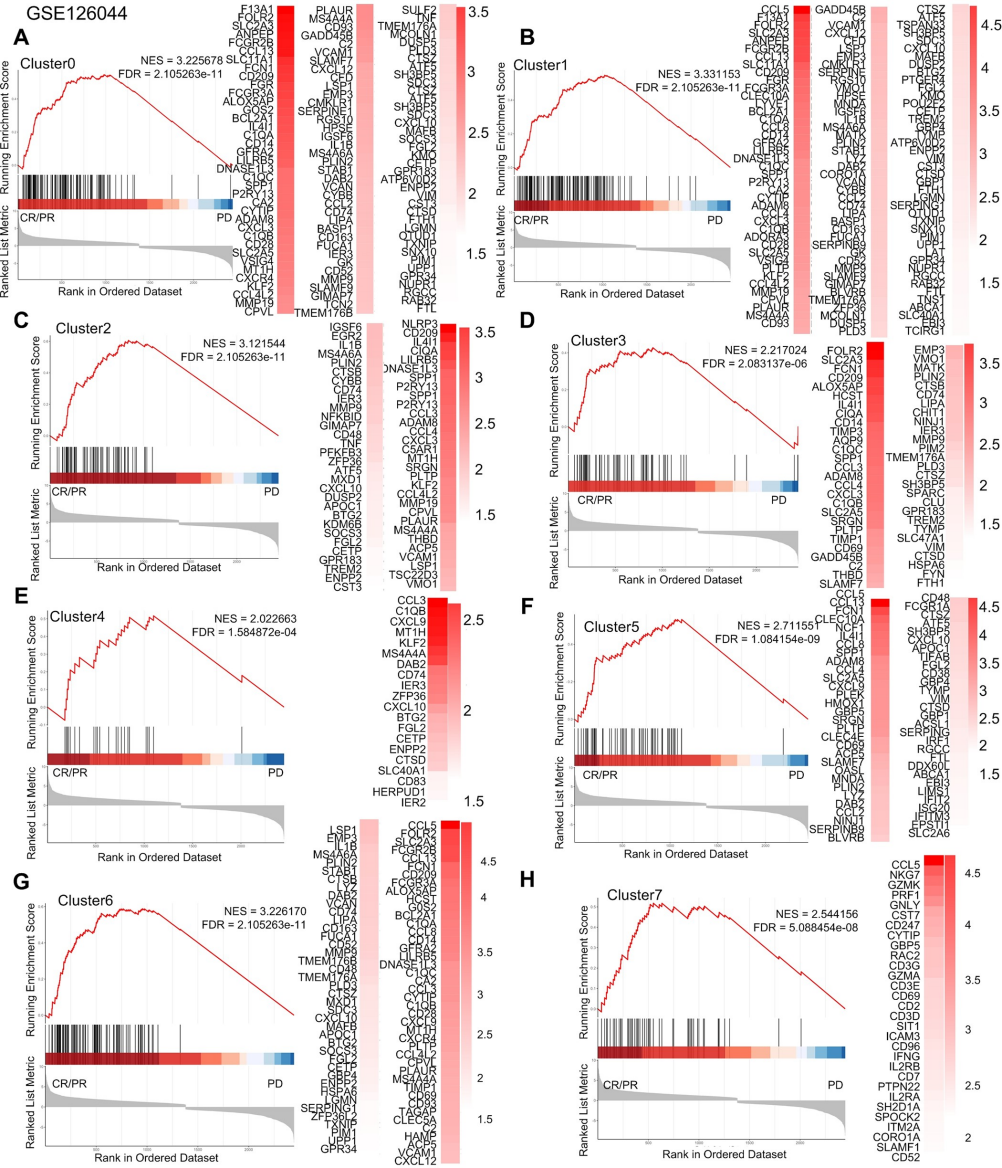
Macrophage subtypes of anti-PD-1 resistance in NSCLC. Gene set enrichment analysis of anti-PD 1 treatment for NSCLC showed that patients in complete remission (CR) and partial remission (PR) compared with those in patients with progressive disease (PD).

**Fig 9:**
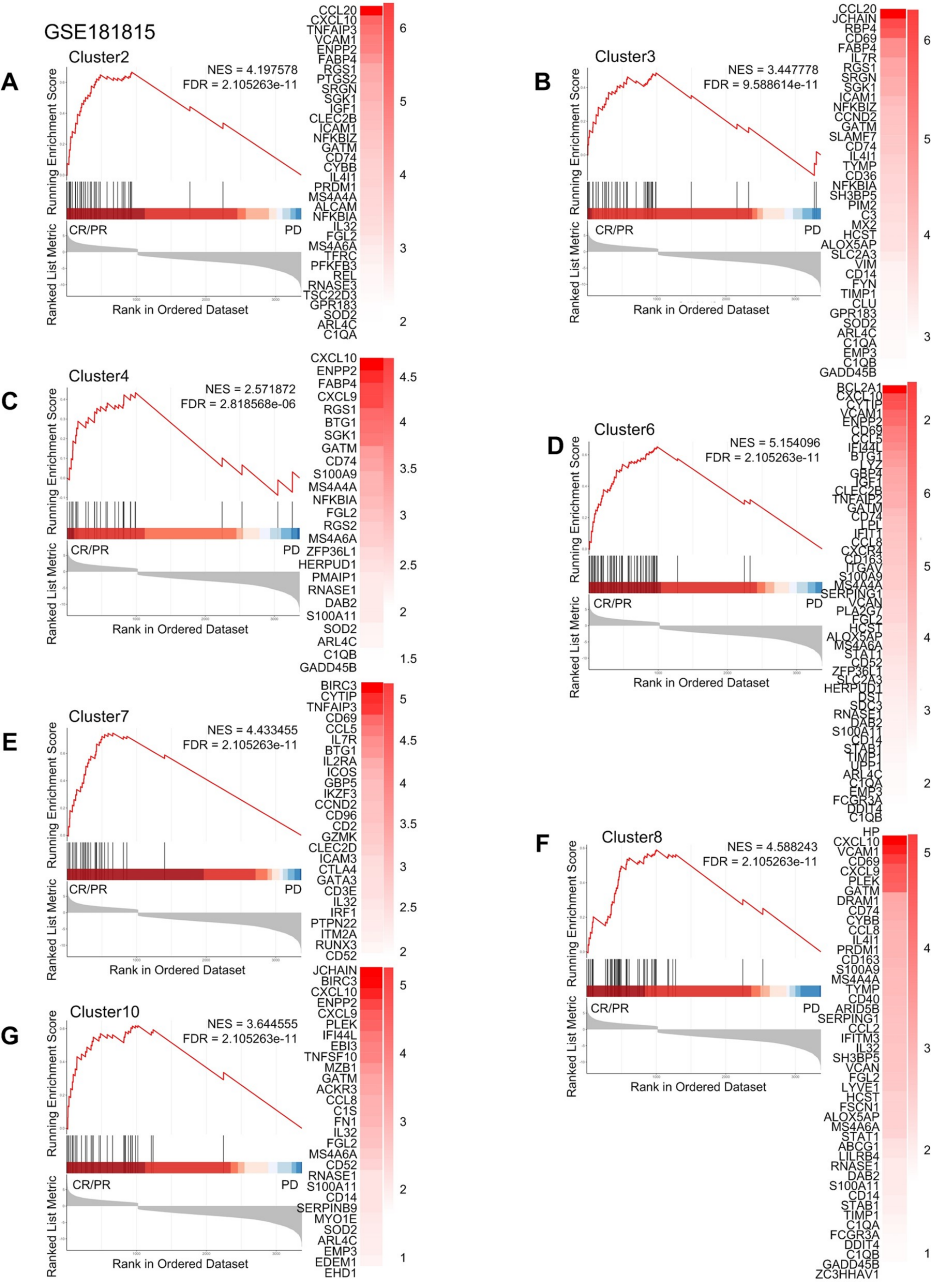
Macrophage subtypes of anti-PD-1 resistance in thymic carcinoma. Gene set enrichment analysis of anti-PD 1 treatment for thymic carcinoma showed that patients in complete remission (CR) and partial remission (PR) compared with those in patients with progressive disease (PD).

Some studies have shown that anti-PD-l immunotherapy may elicit a significant response in patients [31–34]. Furthermore, TAM depletion has been demonstrated to block the PD-l pathway, thereby reactivating infiltrating T cells [35], while different subtypes of TAM exhibit varying sensitivity to anti-PD-1 based immune checkpoint blockade [36]. To investigate the relationship between macrophage subtypes in tumors and the efficacy of immune-targeted therapy, we conducted GSEA analysis using specific signature genes of different macrophage subtypes between patients with progressive disease (PD) and patients with complete response (CR) or partial response (PR) upon anti-PD-1 treatment for melanoma, NSCLC, and thymic carcinoma. We utilized differential expression analysis of different macrophage subtypes to predict treatment sensitivity. Our analyses showed that certain macrophage subtypes in melanoma have gene sets that are positive and significantly enriched in PD when treated with anti-PD-1 (Fig 7A). Conversely, we found positive and significant enrichment of gene sets associated with most macrophage subtypes in NSCLC and liver cancer patients who experienced complete response (CR) or partial response (PR) while receiving anti-PD-1 (Figs 8, 9 and S16). Meanwhile, Cluster9 was not associated with anti-PD-1 drug therapy. These findings suggested that the use of anti-PD-1 drugs affects specific macrophage subpopulations in NSCLC and thymic adenocarcinoma and can ameliorate or treat cancer.

## Discussion

Previous studies have demonstrated that macrophages can polarize into two distinct types, M1 or M2. M1 macrophages are typically induced by Th1 cytokines and are pro-inflammatory cells capable of causing tissue damage as well as having potent anti-tumor activity. M2 macrophages express potent anti-inflammatory cytokine profile and can promote tissue repair but can induce tumor growth and metastasis [37]. In addition, there existed a class of tissue-resident macrophages classified as M2-like, which can act on cell clearance, development, and metabolic regulation, as well as having an M2 anti-inflammatory response [38]. Despite this understanding, the heterogeneity of macrophages in different tumors and its impact on the TME remain largely unknown. Therefore, to gain insight into the diversity of macrophages in pan-cancer, we used scRNA-seq from multiple tumors to characterize macrophages in the TME in the combined set of multiple cancers. Based on differences in gene expression, integrated macrophages can be classified into multiple subtypes that perform different cellular functions and present different prognoses in the TME. These findings may provide valuable insight into intervention strategies targeting macrophages in the treatment of cancer.

Cluster0 and Cluster2 were identified as resident macrophages expressing both anti-inflammatory and pro-inflammatory genes, while Cluster0 showing additional expression of immunomodulators. Moreover, we observed distinct gene expression patterns between these two clusters across different tumors, and there were differences in function between the two clusters. We characterized the expression of tissue-specific resident macrophage signature genes across distinct macrophage subgroups in various cancer types, revealing that Cluster0 and Cluster2 indeed exhibited higher expression levels of tissue-resident macrophage genes. Our study identified the SPP1 pathway as the strongest pathway for TAMs to communicate with each other. This finding echoed previous studies which found that SPP1-positive macrophages play an important role in the TME [30, 39]. Based on these findings, we chose the SPP1 pathway as a focal point for interactions within macrophages. This choice aimed to explore in depth the role and impact of this key pathway among macrophages, providing a unique perspective to better understand macrophage interactions in the TME. The SPP1 signaling pathway was found to be significantly correlated with communication between macrophage subpopulations, with Cluster2 sending signals for intercellular contact with Cluster0. SPP1 encodes the secreted factor osteopontin (OPN), which has extensive immune-related regulatory functions especially in myeloid cells, while OPN proteins undergo extensive post-translational modifications, including phosphorylation, glycosylation and protein hydrolytic cleavage, leading to complex effects [40–42]. Interestingly, both subpopulations showed similar outgoing and incoming patterns, suggesting that they communicate in a similar way and that resident macrophages communicated with each other through the ligand SPP1. Our results showed that except for Cluster8 and Cluster10, all macrophage subpopulations expressed SPP1 genes. SPP1-positive macrophages expressed some genes related to lipid metabolism and myeloid cell activation [43]. Lipid metabolism can regulate the formation and maintenance of tumor stem cells, so targeting lipid metabolism may have some value in the fight against cancer [44]. Previous studies showed that SPP1-CD44 and SPP1-PTGER4 interacted actively with immune cells and mediated crosstalk between tumor cells and macrophages [45, 46] and our study also found that macrophage-to-epithelial cell crosstalk via SPP1-CD44 was also a validation. Also, our study found that SPP1 was differentially expressed in tumors and normal tissues, and in these tumors was positively correlated with the infiltration of immune cells, whereas high expression of SPP1 in some cancers was associated with poor prognosis. Meanwhile, our analysis in tumors with positive correlation between SPP1 and immune infiltration found that most of the SPP1 expression was present with macrophages or monocytes, so in our subsequent analyses, we roughly considered that SPP1 expression in tumors was mainly contributed by macrophages. Macrophages promote immune cell infiltration and release inflammatory cytokines but abnormal production or reactive deleterious activation of macrophages to pro-inflammatory or anti-inflammatory stimuli disrupts tissue homeostasis and promotes disease [47]. Therefore, high expression of SPP1 in macrophages may induce immune responses in tumors and thus affect prognosis. Notably, the characteristic genes of these two subpopulations were highly expressed in KIRC and least expressed in UVM, thus targeting lipid metabolism may be effective in KIRC.

Cluster1 and Cluster3 were identified as TAMs expressing both anti-inflammatory and pro-inflammatory genes. It was found that Cluster1 high expression of metalloproteinase (MMPs), indicating its potential role in disrupting the histological barrier to promote tumor cell invasion, and thus playing a critical role in mid-stage tumor invasion and metastasis. Moreover, the MMP-dependent characteristic of TAMs suggests that a decrease in MMP leads to a decrease in infiltrative TAM, ultimately inhibiting tumor growth [48]. Cluster3 expressed the TGF β coactivator INHBA, which was significantly associated with prognosis in several types of cancer, highlighting the potential prognostic value of Cluster3 in cancer [49–52]. Interestingly, both subpopulations had similar expression levels of transcription factors, which were significantly different from the other subpopulations. When we performed functional analysis of the differentially generated genes in these two clusters, we found that most of the bacterial and viral infection pathways, as well as antigen processing and presentation, were enriched in Cluster3. In the same way, both subpopulations were in the same incoming pattern but not to the same outgoing pattern, indicating that although both belong to TAMs, there were differences in communication with other cells. Notably, high expression of both subpopulations in LGG correlated with poor prognosis, suggesting a strong clinical relevance of TAMs in LGG. Therefore, targeting these two subgroups might provide a potential therapeutic strategy for LGG.

In our study, we observed that Cluster4 was highly expressed in metallothionein gene family. Metallothionein genes played an important role in tumor growth, differentiation, immune escape and drug resistance [53]. Also, we found that Cluster4 exhibited high expression of anti-inflammatory genes relative to other macrophages. Cluster4 was in outgoing pattern II and incoming pattern I, while Cluster5, which showed high expression of pro-inflammatory genes, was classified under outgoing pattern I and incoming pattern II. These findings indicated that different communication patterns may be related to the expression of pro-inflammatory and anti-inflammatory genes.

Both Cluster6 and Cluster8 were characterized by high expression monocyte-like cells markers, indicating that they may be in the process of differentiating from monocytes to macrophages or are just differentiating into macrophages. Cluster6 demonstrated partial anti-inflammatory genes and had intrinsic alveolar macrophage marker PPARG, which may interfere with tumor therapy progression through lipid accumulation and fatty acid synthesis [54, 55]. In contrast, Cluster8 expressed some pro-inflammatory genes, indicating the transformation of monocytes into macrophages with different functions. Despite their different gene expression profiles, both subpopulations share the same pattern of intercellular communication.

In contrast, Cluster7 was found to have high expression levels of anti-inflammatory genes, as well as the cytotoxic gene IL32. Previous studies have shown that IL32β upregulates the production of the anti-inflammatory cytokine IL10, which in turn inhibits the induction of pro-inflammatory cytokines, thereby reducing the inflammatory response [56]. Meanwhile, IL32β has been demonstrated to inhibit tumor growth by activating cytotoxic lymphocyte and inactivating NF-κB and STAT3 pathways through alterations in cytokine levels within the tumor tissue [57]. This also verified the accuracy of the macrophage subpopulation characterization identified in this study.

Our analysis identified a novel macrophage subtype, Cluster9, which showed high expression of genes associated with the cell cycle and S phase compared to other subpopulations. Additionally, Cluster9 expressed higher levels of anti-inflammatory and MMP genes and immunomodulators such as CD209 and CH25H. Among them, blocking CD209 inhibits viral entry [58] and CH25H can suppress viral infection by modulating innate immunity and virus-specific adaptive immunity [59]. Moreover, Cluster9 exhibited high expression of transcription factors E2F2, E2F4, MYCN, and TFDP1, all of which are involved in the regulation of the cell cycle through the p53-p21-RB signaling pathway [60]. These findings suggested that the high expression of proliferating genes by Cluster9 and its tendency to stay in the S phase of the cell cycle could be partially attributed to the regulatory role of these transcription factors.

Cluster10 was highly expressed in selective immunoglobulin genes, indicating a strong association with B cells. Interestingly, these cells were also found to be present in spinal neurons and supraspinal loci, suggesting potential roles during development [61]. These cells also expressed immunomodulators as well as intrinsic alveolar macrophage markers that were capable of performing immune responses against pathogens. Overall, these findings suggested that Cluster10 might play a unique role in the immune system, possibly involving cross-talk with B cells and neuro-immune interactions.

Our analysis showed that some subtypes of macrophages gene sets were significantly enriched in partial response (PR) or complete response (CR) patient groups in both thymic and NSCLCs after anti-PD-1 treatment, suggesting that anti-PD-1 therapy may be an effective approach for tumor immunotherapy. In previous studies, the treatment targeting SPP1+ macrophages in NSCLC with anti-PDL1 has been noted to enhance the progression-free survival of patients [62]. This finding aligned with our results, indicating an association between SPP1 macrophages and improved response of NSCLC to immunotherapy. However, it is important to note that our GSEA results showed that not all macrophages were enriched in sensitive phenotypes when anti-PD-1 therapy was administered to melanoma. This finding was consistent with previous studies, which have shown that PD-1 expression in tumor-draining lymph nodes, but not in tumors, can be associated with the prognosis of melanoma [63]. Therefore, further research is needed to understand the potential differences in the immune microenvironment of different types of tumors and their responses to anti-PD-1 therapy. Similarly, previous findings have demonstrated that human TAMs express elevated levels of PD-1. The expression of PD-1 in TAMs exhibits a negative correlation with their ability to phagocytose tumor cells. Targeting PDL1 has been observed to restore the function of PD-1 TAMs [64, 65]. This suggested that immunotherapy directed at PDL1 could potentially enhance anti-tumor mechanisms.

Our study revealed varying distributions of macrophage subsets across diverse tumors, highlighting the distinct functional properties exhibited by these macrophages in different tumor types (Fig 1F). Notably, our analysis revealed that both Cluster0 and Cluster2 exhibit genes associated with tissue-resident macrophages. These subpopulations were notably prevalent in LIHC, suggesting a potential inclination of LIHC towards the requirement of tissue-resident macrophages. Conversely, Cluster6 and Cluster8, expressing monocyte-like macrophages, exhibited distinct characteristics. Cluster8, prominently represented in lung adenocarcinoma (LUAD), signifies potential functional differences between Cluster6 and Cluster8, potentially influencing their varying proportions in LUAD. Similarly, Cluster1 and Cluster3, expressing genes related to TAMs, displayed contrasting patterns. Cluster3 nearly lacked macrophages in BRCA whereas Cluster1 showed higher macrophage presence in LIHC. These differences in functional traits across subpopulations possibly accounted for their disparate proportions in various cancers.

The present study has several limitations that need to be addressed in future research. Firstly, although we analyzed data from three types of cancer data to construct a pan-cancer macrophage model, it is necessary to analyze more data from different cancers in future studies to validate our findings. Secondly, while transcriptomics is a powerful tool to isolate and analyze different macrophage subpopulations, this method should be complemented with other techniques such as fluorescence-activated cell sorting and immunohistochemistry to better characterize macrophages and identify marker genes between different subpopulations. In addition, for the association between drug resistance and macrophage subpopulation, we only analysed the resistance to anti-PD-1 therapies. In future study, the analysis of other immunotherapy treatments needed to be performed to comprehensively confirm the correlation between macrophage subpopulations and immunotherapy resistance. Furthermore, our study focused on transcriptomic data of macrophages, however, previous research has shown that genome wide analysis, differentially methylated regions are associated with anti-PD-1 efficacy [66]. Therefore, it is also important to explore the association between the genomic data of macrophages and anti-PD-1 efficacy.

In summary, this study provides a comprehensive and systematic overview of 11 subtypes of macrophages classified in multiple cancers. By analysing the interrelationship between different macrophages, we gained insights into how different macrophages communicate with each other and how they can affect subsequent treatment outcomes. The global structure of UMAP is also reflected in the similarity between macrophage subtypes [67]. Moreover, our findings suggested that targeting differences in macrophages and changing the immune microenvironment may be a promising treatment for cancer. Future studies on drug resistance of different macrophage subtypes can evaluate the function and outcome of therapies of drug-resistant tumor. The current study outlined the functions and differences between the different subtypes, providing a basis for further clinical relevance studies.

## Materials and methods

### Cell lines and cell culture

The THP-1 human monocytic cell line, MDA-MB 231 breast cancer cell lines, A549 lung cancer cell lines, HepG2 liver cancer cell lines were obtained from the American Type Culture Collection (ATCC). THP-1 was maintained in RPMI 1640 (Life Technologies) supplemented with 10% fetal bovine serum (FBS, Life Technologies), 1% penicillin/streptomycin and 0.05 mM 2-mercaptoethanol (Sigma). MDA-MB 231, A549 and HepG2 was maintained in DMEM (Life Technologies) supplemented with 10% fetal bovine serum (Life Technologies). To generate adherent THP-1-derived macrophages, 1 × 10^6^ cells were added to wells in an untreated TCP six-well plate (Becton Dickinson) and treated with 20 nmol/L of phorbol myristate acetate (PMA; Sigma) dissolved in media for 48 h at 37°C, 5% CO2. Macrophage was confirmed using flow cytometry by detecting CD14, and for the purposes of this study, will be considered as having an M0 phenotype. For TAM generation, in co-culture system, macrophages were co-cultured with cancer cells or condition medium from cancer cells to generate TAMs. Macrophages were seeded in lower inserts of 6-well transwell plate [0.4 μm pore size polycarbonate transwell filters (Life Sciences)], and cancer cells were seeded in upper inserts. The two cells were co-cultured without direct contact. After 48 h, the macrophages in the lower inserts were used for next treatment. Tumor cells and macrophages were treated with rh SPP1 (MedChemExpress) and anti-SPP1 antibody (R&D).

### Plasmids and lenti-virus infection

The SPP1 expression vector was constructed into the pLVPT plasmid. Lentivirus including human SPP1 was produced in 293T cells. THP-1 cell lines with vector and stable SPP1 overexpression were constructed by infecting lentivirus as previously described [68].

### Western blotting

After being washed three times by PBS, cells were harvested and solubilized in cold radioimmunoprecipitation assay (RIPA) lysis buffer containing phenylmethylsulfonyl fluoride (PMSF) and protease inhibitor cocktail. Total protein was quantified using a BCA protein assay. Equal amounts of each protein sample (30 μg) were separated on 10–15% sodium dodecyl sulfate polyacrylamide and transferred to a polyvinylidene fluoride (PVDF) membrane for 1–1.5 h. The membranes were blocked with 5% milk or BSA in Tris-buffered saline with Tween (TBST), the membranes were incubated with primary rabbit antibodies to anti-osteopontin antibody (1:1000, ab214050, Abcam) or anti-ACTB (1:1000, 6487S, Cell Signaling Technology) at 4°C overnight. Subsequently, the membrane was washed with TBST three times (5 mins each) and incubated with secondary anti-rabbit antibody to IgG (1:2000, 7074S, Cell Signaling Technology) at room temperature for 2 h. After washing, the protein signals on the membranes were visualized with enhanced chemiluminescence (ECL) substrate in a FluorChem Q imaging system.

### Enzyme-linked immunosorbent assay (ELISA)

The plasma SPP1, TGF β, IL10 and VEGF levels in media (FBS-free) were assayed using a commercial enzyme-linked immunosorbent assay (ELISA) kit (ELH-OPN-1; Abcam). The 25-fold diluted plasma samples were directly transferred to a 96-well plate coated with an antibody specific for human SPP1, assay buffer was added to each well, then the test sample and the control sample were added to the corresponding wells and incubated at room temperature for 2 h. The wells were washed and the detection antibody solution was added, and incubated for another 1 h at room temperature. After washing once using PBS, the enzyme-labeled secondary antibody was added for 30 min, the developer was added in the dark for 15 min, and then the stop solution was added. The absorbance value was recorded at 450 nm in a microtest plate spectrophotometer with the correction wavelength set at 540 nm. SPP1 levels were quantified using a calibration curve based on a human osteopontin standard. Both standards and samples were evaluated in duplicate, and the results were adopted only when the inter-assay variations were within the range provided by the manufacturer.

### Data acquisition and preprocessing

We used single-cell data downloaded from the Gene Expression Omnibus (GEO) database for breast cancer (GSE148673), liver cancer (GSE166635), and lung adenocarcinoma (GSE171145). The selection of specific single-cell datasets we based on the following three considerations. Firstly, we prioritized Illumina NovaSeq 6000 (Homo sapiens), a unified sequencing platform, to minimize the impact on the results due to the errors associated with sequencing with different instrumentation. Secondly, this platform in the GEO database provides a wide range of resources, and we purposely selected data from recent years to reduce the batch-to-batch variation between different tumor datasets and to ensure the consistency and comparability of the data. Single-cell sequencing technologies and platforms have undergone significant development and improvement over the past few years, so the selection of recent data allows for the use of more technologically stable and advanced data, and reduces the impact of possible technological differences on the results. Finally, single-cell sequencing data for different types of cancers were selected with the goal of comparing and analyzing the differences and commonalities between these tumors. Different types of cancers may differ in their pathophysiology and immune environments, and by understanding the commonalities and differences in macrophages across cancer types, it helps to develop an understanding of pan-cancer traits, which are shared biological characteristics across multiple cancer types. For these reasons, we found single-cell sequencing data from only three of the above tumors eligible for analysis. We integrated all datasets using a "merge" technique designed to fully integrate the information. We then reduced batch effects using the canonical correlation analysis (CCA) method [69, 70]. The "CCA" method is a method that comes with the Seruat package and eliminates technical bias by constituting paired anchor cells. Additionally, we downloaded a total of 33 datasets of all cancers from The Cancer Genome Atlas (TCGA) by utilizing the TCGAbiolinks package (v 2.25.3). To analyze the TCGA data, we employed the Transcripts Per Kilobase of exon model per Million mapped reads (TPM) method.

### Single Cell RNA-seq analysis of macrophages

We used the data in the public database to extract the macrophage types by the Seurat (v 4.3.0) to perform a detailed cluster analysis of each macrophage in cancer separately. Cells with the number of expressing genes below 200 or above 6000 were removed. In addition, Cells with higher than 10% mitochondrial gene content were removed prior to further analysis. Visualized the images by dimensionality reduction (UMAP), and annotated them according to different clusters. To compare and identify macrophage subpopulations between different tumor types, datasets from BRCA, LIHC, and lung cancer (LUAD) were integrated by canonical correlation analysis (CCA). We used KEGG and Gene Ontology (GO)—Biological Process, to analyze macrophage function in different tumors as well as macrophage function after integration, respectively, and when the enrichment for pathway was considered significant at p < 0.05. To assess the differences in transcription factors between subpopulations, we used DoRothEA (v 1.6.0) to analyze them based on single-cell RNA data.

### Cell communication analysis

We used CellChat (v 1.5.0) [71], a network analysis and pattern recognition tool, to analyze the single-cell RNA-sequencing data of macrophages. Cellular communication analysis involved the use of gene expression data to infer communication between cells based on the network analysis and pattern recognition methods provided by CellChat. We used CellChat to analyze single-cell RNA data of macrophages to obtain the strength and number of information interactions between various subpopulations. Moreover, we calculated the strongest information interaction pathway SPP1 and performed further analysis, and finally obtained the communication between the senders and receivers in the SPP1 pathway.

### TCGA data analysis

We downloaded TCGA data using the TCGAbiolinks package [72]. All the tumors with significant differences in SPP1 in tumor and normal tissues were analyzed by single-sample gene set enrichment analysis (ssGSEA) immune infiltration, and the correlation between SPP1 expression and different degrees of immune cell infiltration was analyzed by Hmisc package (v 5.1.0). We performed spearman correlation analysis on the genes of CD44, ITGB1, and ITGB6 in 33 cancers, and when the correlation was higher than 0.3 and the pvalue was below 0.05 as related genes, KEGG enrichment analysis was performed. The intersection of the enriched pathways in different cancers was demonstrated using the UpSetR package (v1.4.0). We performed prognostic analysis of macrophage subpopulations in pan-cancer by defining the signature genes for each subpopulation. We screened the genes that differed from other subpopulations of cells in the three cancer types BRCA, LIHC, and LUAD by the FindMarkers function in Seurat. To ensure significance of the differential genes, we setted genes with adjust.P < 0.05 and log_2_FoldChange > 0.3 as specific signature genes. The expression of these macrophage signature genes was extracted from the TPM values of 33 cancer types RNA data from the TCGA database, and the expression of each subgroup of signature genes was obtained by averaging all the signature genes after Z-score calculation. We represented the overall expression of the signature genes in tumor samples by averaging all the signature genes after Z-score calculation. After sorting the different cancer types of different signature genes, we selected the top 1/4 and bottom 1/4 samples for survival analysis. The p-values were converted to false discovery rate (FDR) by R (v 4.1.3), and FDR < 0.05 cancers were screened for subsequent analysis. In addition, we conducted multivariate analysis for each cancer, adjusting for age and gender as continuous and dichotomous variables, respectively. We conducted univariate analysis for all cancers and calculated FDR values, with FDR < 0.05 being statistically significant.

### Gene Set Enrichment Analysis (GSEA)

We performed GSEA in cancer types with significant survival differences between high and low expression signature genes of different macrophage subpopulations. We used edgeR and Wilcoxon rank sum tests to obtain differential expression between the first and last 1/4 samples. The gene set of differential genes in the REACTOME database were analyzed, with significance adjust. P < 0.05. We used different colors to express enrichment or depletion in cancer, and results of the selection of enrichment scores in the top 10 were selected separately in order to reduce the results with smaller effects.

### Immunotherapy sensitivity and resistance analysis

We selected data from melanoma (GSE78220) [73], NSCLC (GSE126044)[66], and thymic carcinoma (GSE181815)[74] after anti-PD-1 treatment. We performed enrichment analysis of the marker gene sets of each subgroup by the GSEA to analyze their significant enrichment between the therapeutically resistant or sensitive.

## Supporting information

S1 Fig Macrophages in different cancer types. (A) UMAP distribution, expression of marker genes and gene enrichment analysis of macrophages in breast cancer. (B) Distribution of UMAP, expression of marker genes, and gene enrichment analysis of macrophages in hepatocellular carcinoma. (C) Distribution of UMAP, expression of marker genes, and gene enrichment analysis of macrophages in lung cancer.
(TIF)

S2 Fig Differential gene and functional analysis of similarly expressed subpopulations. Differential gene volcano maps for different cancer types in Cluster0(A) and Cluster2 (B) (tissue-resident macrophages). (C) Expression of cancer-specific resident macrophage gene sets in different subpopulations.
(TIF)

S3 Fig Functional analysis of similarly expressed subpopulations. Functional differences between similar subgroups, (A) Cluster0 vs Cluster2, (B) Cluster1 vs Cluster3, (C) Cluster6 vs Cluster8.
(TIF)

S4 Fig Cellular communication between macrophage subtypes. (A) The number of intercellular communications in different subpopulations, the greater the number, the thicker the linkage between different subgroups. (B) Intercellular communication strength of different subpopulations, the higher the intensity, the thicker the linkage between subgroups. (C) The communication patterns of intercellular inputs of different cell subpopulations. (D) The communication patterns of intercellular outgoings of different cell subpopulations.
(TIF)

S5 Fig Relative expression of SPP1 in each cell type in different cancers. (A) UMAP distribution of cell types of THCA and UMAP showing SPP1 expression. (B) UMAP distribution of cell types of COAD and UMAP showing SPP1 expression. (C) UMAP distribution of cell types of PRAD and UMAP showing SPP1 expression
(TIF)

S6 Fig Prognostic effects of SPP1 in different cancers. The light-colored background area is the confidence interval of the probability of survival at each time point calculated by the KM method, which is the 95% confidence interval, Time(D) = Time (Days).
(TIF)

S7 Fig Enrichment pathways of different receptors in different cancers. Intersection of enrichment pathways for different receptors in 33 cancers, CD44(A), ITGB1(B).
(TIF)

S8 Fig ITGB6 receptor enrichment pathways in different cancers. Intersection of ITGB6 receptor enrichment pathways in 33 cancers.
(TIF)

S9 Fig Expression of different subgroups (Cluster0-2) of characteristic genes in pan-cancer. Expression of genes characteristic of macrophage subpopulations in 33 tumors in descending order, Cluster0 (A), Cluster1 (B), Cluster2 (C).
(TIF)

S10 Fig Expression of different subgroups (Cluster3-5) of characteristic genes in pan-cancer. Expression of genes characteristic of macrophage subpopulations in 33 tumors in descending order, Cluster3 (A), Cluster4 (B), Cluster5 (C).
(TIF)

S11 Fig Expression of different subgroups (Cluster6-8) of characteristic genes in pan-cancer. Expression of genes characteristic of macrophage subpopulations in 33 tumors in descending order, Cluster6 (A), Cluster7 (B), Cluster8 (C).
(TIF)

S12 Fig Expression of different subgroups (Cluster9-10) of characteristic genes in pan-cancer. Expression of genes characteristic of macrophage subpopulations in 33 tumors in descending order, Cluster9 (A), Cluster10 (B).
(TIF)

S13 Fig Tumor types with a better prognosis for characteristic genes. Kaplan-Meier curves depicting survival differences in high and low expression signature genes in multiple subpopulations for THYM (A), and SKCM (B-K), BRCA (L), UCEC (M). The light-colored background area is the confidence interval of the probability of survival at each time point calculated by the KM method, which is the 95% confidence interval, Time(D) = Time (Days).
(TIF)

S14 Fig Networks of REACTOME terms enriched or depleted in tumors with high expression of different macrophage subtypes (Cluster4-Cluster7) signature genes. (A) Results of THYM, SKCM and LGG for Cluster4. (B) Results of PAAD, LGG, SKCM, THYM on Cluster5. (C) Results of SKCM, LGG, GBM in Cluster6. (D) Results of UCEC, BRCA, SKCM, LGG in Cluster7.
(TIF)

S15 Fig Networks of REACTOME terms enriched or depleted in tumors with high expression of different macrophage subtypes (Cluster8-Cluster10) signature genes. (A) Results of SKCM, LGG in Cluster8. (B) Results of LUAD, PAAD, KIRP, THYM, MESO, LGG, ACC, UCEC in Cluster 9. (C) Results for SKCM, LGG in Cluster10.
(TIF)

S16 Fig Macrophage subtypes (Cluster8, Cluster10) in anti-PD-1 resistant NSCLC. Gene set enrichment analysis of anti-PD 1 treatment for NSCLC of Cluster8 and Cluster10 showed that patients in complete remission (CR) and partial remission (PR) compared with those in patients with progressive disease (PD).
(TIF)

S1 Table Relationship between the number of different macrophage subtypes in a single cancer. (XLSX)

S2 Table Signaling pathways between different macrophage subpopulations and corresponding strengths. (XLSX)

S3 Table Signaling pathways and corresponding strengths between epithelial cells and macrophages. (XLSX)

S4 Table Multivariate Cox proportional risk modeling analysis of individual macrophage subtype gene signatures while adjusting for age and gender. (XLSX)

## References

[1] Cheng YQ, Wang SB, Liu JH, Jin L, Liu Y, Li CY, et al. Modifying the tumour microenvironment and reverting tumour cells: New strategies for treating malignant tumours. Cell Prolif. 2020;53(8):e12865. doi: 10.1111/cpr.12865 32588948 PMC7445401

[2] Jahchan NS, Mujal AM, Pollack JL, Binnewies M, Sriram V, Reyno L, et al. Tuning the Tumor Myeloid Microenvironment to Fight Cancer. Front Immunol. 2019;10:1611. doi: 10.3389/fimmu.2019.01611 31402908 PMC6673698

[3] Chen D, Zhang X, Li Z, Zhu B. Metabolic regulatory crosstalk between tumor microenvironment and tumor-associated macrophages. Theranostics. 2021;11(3):1016–30. doi: 10.7150/thno.51777 33391518 PMC7738889

[4] Dancsok AR, Gao D, Lee AF, Steigen SE, Blay JY, Thomas DM, et al. Tumor-associated macrophages and macrophage-related immune checkpoint expression in sarcomas. Oncoimmunology. 2020;9(1):1747340. doi: 10.1080/2162402X.2020.1747340 32313727 PMC7153829

[5] Hill DA, Lim HW, Kim YH, Ho WY, Foong YH, Nelson VL, et al. Distinct macrophage populations direct inflammatory versus physiological changes in adipose tissue. Proc Natl Acad Sci U S A. 2018;115(22):E5096–E105. doi: 10.1073/pnas.1802611115 29760084 PMC5984532

[6] Artyomov MN, Sergushichev A, Schilling JD. Integrating immunometabolism and macrophage diversity. Semin Immunol. 2016;28(5):417–24. doi: 10.1016/j.smim.2016.10.004 27771140 PMC5333784

[7] Locati M, Curtale G, Mantovani A. Diversity, Mechanisms, and Significance of Macrophage Plasticity. Annu Rev Pathol. 2020;15:123–47. doi: 10.1146/annurev-pathmechdis-012418-012718 31530089 PMC7176483

[8] Kadomoto S, Izumi K, Mizokami A. Macrophage Polarity and Disease Control. Int J Mol Sci. 2021;23(1). doi: 10.3390/ijms23010144 35008577 PMC8745226

[9] Wynn TA, Chawla A, Pollard JW. Macrophage biology in development, homeostasis and disease. Nature. 2013;496(7446):445–55. doi: 10.1038/nature12034 23619691 PMC3725458

[10] Yunna C, Mengru H, Lei W, Weidong C. Macrophage M1/M2 polarization. Eur J Pharmacol. 2020;877:173090. doi: 10.1016/j.ejphar.2020.173090 32234529

[11] Mantovani A, Sozzani S, Locati M, Allavena P, Sica A. Macrophage polarization: tumor-associated macrophages as a paradigm for polarized M2 mononuclear phagocytes. Trends Immunol. 2002;23(11):549–55. doi: 10.1016/s1471-4906(02)02302-5 12401408

[12] Saradna A, Do DC, Kumar S, Fu QL, Gao P. Macrophage polarization and allergic asthma. Transl Res. 2018;191:1–14. doi: 10.1016/j.trsl.2017.09.002 29066321 PMC5776696

[13] Spiller KL, Nassiri S, Witherel CE, Anfang RR, Ng J, Nakazawa KR, et al. Sequential delivery of immunomodulatory cytokines to facilitate the M1-to-M2 transition of macrophages and enhance vascularization of bone scaffolds. Biomaterials. 2015;37:194–207. doi: 10.1016/j.biomaterials.2014.10.017 25453950 PMC4312192

[14] Alvarez MM, Liu JC, Trujillo-de Santiago G, Cha BH, Vishwakarma A, Ghaemmaghami AM, et al. Delivery strategies to control inflammatory response: Modulating M1-M2 polarization in tissue engineering applications. J Control Release. 2016;240:349–63. doi: 10.1016/j.jconrel.2016.01.026 26778695 PMC4945478

[15] Chamseddine AN, Assi T, Mir O, Chouaib S. Modulating tumor-associated macrophages to enhance the efficacy of immune checkpoint inhibitors: A TAM-pting approach. Pharmacol Ther. 2022;231:107986. doi: 10.1016/j.pharmthera.2021.107986 34481812

[16] Ehlers FAI, Mahaweni NM, Berends AV, Saya T, Bos GMJ, Wieten L. Exploring the potential of combining IL-2-activated NK cells with an anti-PDL1 monoclonal antibody to target multiple myeloma-associated macrophages. Cancer Immunol Immun. 2023;72(6):1789–801. doi: 10.1007/s00262-022-03365-4 36656341 PMC10198883

[17] Ngambenjawong C, Gustafson HH, Pun SH. Progress in tumor-associated macrophage (TAM)-targeted therapeutics. Adv Drug Deliv Rev. 2017;114:206–21. doi: 10.1016/j.addr.2017.04.010 28449873 PMC5581987

[18] Li X, Wang CY. From bulk, single-cell to spatial RNA sequencing. Int J Oral Sci. 2021;13(1):36. doi: 10.1038/s41368-021-00146-0 34782601 PMC8593179

[19] Kolodziejczyk AA, Kim JK, Svensson V, Marioni JC, Teichmann SA. The technology and biology of single-cell RNA sequencing. Mol Cell. 2015;58(4):610–20. doi: 10.1016/j.molcel.2015.04.005 26000846

[20] Jovic D, Liang X, Zeng H, Lin L, Xu F, Luo Y. Single-cell RNA sequencing technologies and applications: A brief overview. Clin Transl Med. 2022;12(3):e694. doi: 10.1002/ctm2.694 35352511 PMC8964935

[21] Ochocka N, Segit P, Walentynowicz KA, Wojnicki K, Cyranowski S, Swatler J, et al. Single-cell RNA sequencing reveals functional heterogeneity of glioma-associated brain macrophages. Nat Commun. 2021;12(1):1151. doi: 10.1038/s41467-021-21407-w 33608526 PMC7895824

[22] MacParland SA, Liu JC, Ma XZ, Innes BT, Bartczak AM, Gage BK, et al. Single cell RNA sequencing of human liver reveals distinct intrahepatic macrophage populations. Nat Commun. 2018;9(1):4383. doi: 10.1038/s41467-018-06318-7 30348985 PMC6197289

[23] Yang Q, Zhang H, Wei T, Lin A, Sun Y, Luo P, et al. Single-Cell RNA Sequencing Reveals the Heterogeneity of Tumor-Associated Macrophage in Non-Small Cell Lung Cancer and Differences Between Sexes. Front Immunol. 2021;12:756722. doi: 10.3389/fimmu.2021.756722 34804043 PMC8602907

[24] Ho DW, Tsui YM, Chan LK, Sze KM, Zhang X, Cheu JW, et al. Single-cell RNA sequencing shows the immunosuppressive landscape and tumor heterogeneity of HBV-associated hepatocellular carcinoma. Nat Commun. 2021;12(1):3684. doi: 10.1038/s41467-021-24010-1 34140495 PMC8211687

[25] Zhang L, Li Z, Skrzypczynska KM, Fang Q, Zhang W, O’Brien SA, et al. Single-Cell Analyses Inform Mechanisms of Myeloid-Targeted Therapies in Colon Cancer. Cell. 2020;181(2):442–59 e29. doi: 10.1016/j.cell.2020.03.048 32302573

[26] Ramos RN, Missolo-Koussou Y, Gerber-Ferder Y, Bromley CP, Bugatti M, Núñez NG, et al. Tissue-resident FOLR2 macrophages associate with CD8 T cell infiltration in human breast cancer. Cell. 2022;185(7):1189-+. doi: 10.1016/j.cell.2022.02.021 35325594

[27] Sierro F, Evrard M, Rizzetto S, Melino M, Mitchell AJ, Florido M, et al. A Liver Capsular Network of Monocyte-Derived Macrophages Restricts Hepatic Dissemination of Intraperitoneal Bacteria by Neutrophil Recruitment. Immunity. 2017;47(2):374-+. doi: 10.1016/j.immuni.2017.07.018 28813662

[28] Xiang C, Zhang M, Shang ZX, Chen SN, Zhao JK, Ding BW, et al. Single-cell profiling reveals the trajectory of FOLR2-expressing tumor-associated macrophages to regulatory T cells in the progression of lung adenocarcinoma. Cell Death & Disease. 2023;14(8). ARTN 493. doi: 10.1038/s41419-023-06021-6 37532692 PMC10397223

[29] Qian J, Olbrecht S, Boeckx B, Vos H, Laoui D, Etlioglu E, et al. A pan-cancer blueprint of the heterogeneous tumor microenvironment revealed by single-cell profiling. Cell Res. 2020;30(9):745–62. doi: 10.1038/s41422-020-0355-0 32561858 PMC7608385

[30] Bill R, Wirapati P, Messemaker M, Roh W, Zitti B, Duval F, et al. CXCL9:SPP1 macrophage polarity identifies a network of cellular programs that control human cancers. Science. 2023;381(6657):515–24. doi: 10.1126/science.ade2292 37535729 PMC10755760

[31] Bhave P, Ahmed T, Lo SN, Shoushtari A, Zaremba A, Versluis JM, et al. Efficacy of anti-PD-1 and ipilimumab alone or in combination in acral melanoma. J Immunother Cancer. 2022;10(7). doi: 10.1136/jitc-2022-004668 35793872 PMC9260790

[32] Zheng Y, Wang T, Tu X, Huang Y, Zhang H, Tan D, et al. Gut microbiome affects the response to anti-PD-1 immunotherapy in patients with hepatocellular carcinoma. J Immunother Cancer. 2019;7(1):193. doi: 10.1186/s40425-019-0650-9 31337439 PMC6651993

[33] Pingili AK, Chaib M, Sipe LM, Miller EJ, Teng B, Sharma R, et al. Immune checkpoint blockade reprograms systemic immune landscape and tumor microenvironment in obesity-associated breast cancer. Cell Rep. 2021;35(12):109285. doi: 10.1016/j.celrep.2021.109285 34161764 PMC8574993

[34] Yan J, Chen Y, Luo M, Hu X, Li H, Liu Q, et al. Chronic stress in solid tumor development: from mechanisms to interventions. J Biomed Sci. 2023;30(1):8. doi: 10.1186/s12929-023-00903-9 36707854 PMC9883141

[35] Li Z, Ding Y, Liu J, Wang J, Mo F, Wang Y, et al. Depletion of tumor associated macrophages enhances local and systemic platelet-mediated anti-PD-1 delivery for post-surgery tumor recurrence treatment. Nat Commun. 2022;13(1):1845. doi: 10.1038/s41467-022-29388-0 35387972 PMC8987059

[36] Zhang H, Liu Z, Wen H, Guo Y, Xu F, Zhu Q, et al. Immunosuppressive TREM2(+) macrophages are associated with undesirable prognosis and responses to anti-PD-1 immunotherapy in non-small cell lung cancer. Cancer Immunol Immunother. 2022;71(10):2511–22. doi: 10.1007/s00262-022-03173-w 35278107 PMC10991123

[37] Shapouri-Moghaddam A, Mohammadian S, Vazini H, Taghadosi M, Esmaeili SA, Mardani F, et al. Macrophage plasticity, polarization, and function in health and disease. J Cell Physiol. 2018;233(9):6425–40. doi: 10.1002/jcp.26429 29319160 PMC13482560

[38] Davies LC, Jenkins SJ, Allen JE, Taylor PR. Tissue-resident macrophages. Nat Immunol. 2013;14(10):986–95. doi: 10.1038/ni.2705 24048120 PMC4045180

[39] Han H, Ge X, Komakula SSB, Desert R, Das S, Song Z, et al. Macrophage-derived Osteopontin (SPP1) Protects From Nonalcoholic Steatohepatitis. Gastroenterology. 2023;165(1):201–17. doi: 10.1053/j.gastro.2023.03.228 37028770 PMC10986640

[40] Shen XT, Xie SZ, Xu J, Yang LY, Qin LX. Pan-Cancer Analysis Reveals a Distinct Neutrophil Extracellular Trap-Associated Regulatory Pattern. Front Immunol. 2022;13:798022. doi: 10.3389/fimmu.2022.798022 35432310 PMC9009150

[41] Xu K, Tian X, Oh SY, Movassaghi M, Naber SP, Kuperwasser C, et al. The fibroblast Tiam1-osteopontin pathway modulates breast cancer invasion and metastasis. Breast Cancer Res. 2016;18(1):14. doi: 10.1186/s13058-016-0674-8 26821678 PMC4730665

[42] Cheng YR, Li Y, Scherer N, Grundner-Culemann F, Lehtimäki T, Mishra BH, et al. Genetics of osteopontin in patients with chronic kidney disease: The German chronic kidney disease study. Plos Genet. 2022;18(4). ARTN e1010139. doi: 10.1371/journal.pgen.1010139 35385482 PMC9015153

[43] Papazoglou A, Huang M, Bulik M, Lafyatis A, Tabib T, Morse C, et al. Epigenetic Regulation of Profibrotic Macrophages in Systemic Sclerosis-Associated Interstitial Lung Disease. Arthritis Rheumatol. 2022;74(12):2003–14. doi: 10.1002/art.42286 35849803 PMC9771864

[44] Li H, Feng Z, He ML. Lipid metabolism alteration contributes to and maintains the properties of cancer stem cells. Theranostics. 2020;10(16):7053–69. doi: 10.7150/thno.41388 32641978 PMC7330842

[45] Wang CD, Yu QX, Song TT, Wang ZF, Song LJ, Yang Y, et al. The heterogeneous immune landscape between lung adenocarcinoma and squamous carcinoma revealed by single-cell RNA sequencing. Signal Transduct Tar. 2022;7(1). ARTN 289. doi: 10.1038/s41392-022-01130-8 36008393 PMC9411197

[46] Liu L, Zhang R, Deng J, Dai X, Zhu X, Fu Q, et al. Construction of TME and Identification of crosstalk between malignant cells and macrophages by SPP1 in hepatocellular carcinoma. Cancer Immunol Immunother. 2022;71(1):121–36. doi: 10.1007/s00262-021-02967-8 34028567 PMC10992184

[47] Greene JT, Brian BFt, Senevirathne SE, Freedman TS. Regulation of myeloid-cell activation. Curr Opin Immunol. 2021;73:34–42. doi: 10.1016/j.coi.2021.09.004 34601225 PMC8648988

[48] Gui P, Ben-Neji M, Belozertseva E, Dalenc F, Franchet C, Gilhodes J, et al. The Protease-Dependent Mesenchymal Migration of Tumor-Associated Macrophages as a Target in Cancer Immunotherapy. Cancer Immunol Res. 2018;6(11):1337–51. doi: 10.1158/2326-6066.CIR-17-0746 30181209

[49] Li X, Yu W, Liang C, Xu Y, Zhang M, Ding X, et al. INHBA is a prognostic predictor for patients with colon adenocarcinoma. BMC Cancer. 2020;20(1):305. doi: 10.1186/s12885-020-06743-2 32293338 PMC7161248

[50] Liu M, Smith R, Liby T, Chiotti K, Lopez CS, Korkola JE. INHBA is a mediator of aggressive tumor behavior in HER2+ basal breast cancer. Breast Cancer Res. 2022;24(1):18. doi: 10.1186/s13058-022-01512-4 35248133 PMC8898494

[51] Zhao K, Yi Y, Ma Z, Zhang W. INHBA is a Prognostic Biomarker and Correlated With Immune Cell Infiltration in Cervical Cancer. Front Genet. 2021;12:705512. doi: 10.3389/fgene.2021.705512 35058963 PMC8764128

[52] Ojeda-Fernandez L, Recio-Poveda L, Aristorena M, Lastres P, Blanco FJ, Sanz-Rodriguez F, et al. Mice Lacking Endoglin in Macrophages Show an Impaired Immune Response. Plos Genet. 2016;12(3):e1005935. doi: 10.1371/journal.pgen.1005935 27010826 PMC4806930

[53] Si M, Lang J. The roles of metallothioneins in carcinogenesis. J Hematol Oncol. 2018;11(1):107. doi: 10.1186/s13045-018-0645-x 30139373 PMC6108115

[54] Ma S, Zhou B, Yang Q, Pan Y, Yang W, Freedland SJ, et al. A Transcriptional Regulatory Loop of Master Regulator Transcription Factors, PPARG, and Fatty Acid Synthesis Promotes Esophageal Adenocarcinoma. Cancer Res. 2021;81(5):1216–29. doi: 10.1158/0008-5472.CAN-20-0652 33402390 PMC8026506

[55] Wilson HE, Stanton DA, Rellick S, Geldenhuys W, Pistilli EE. Breast cancer-associated skeletal muscle mitochondrial dysfunction and lipid accumulation is reversed by PPARG. Am J Physiol Cell Physiol. 2021;320(4):C577–C90. doi: 10.1152/ajpcell.00264.2020 33439777 PMC8260354

[56] Kang JW, Choi SC, Cho MC, Kim HJ, Kim JH, Lim JS, et al. A proinflammatory cytokine interleukin-32beta promotes the production of an anti-inflammatory cytokine interleukin-10. Immunology. 2009;128(1 Suppl):e532-40. doi: 10.1111/j.1365-2567.2008.03025.x 19740314 PMC2753893

[57] Yun HM, Oh JH, Shim JH, Ban JO, Park KR, Kim JH, et al. Antitumor activity of IL-32beta through the activation of lymphocytes, and the inactivation of NF-kappaB and STAT3 signals. Cell Death Dis. 2013;4(5):e640. doi: 10.1038/cddis.2013.166 23703385 PMC3674373

[58] Amraei R, Yin W, Napoleon MA, Suder EL, Berrigan J, Zhao Q, et al. CD209L/L-SIGN and CD209/DC-SIGN Act as Receptors for SARS-CoV-2. ACS Cent Sci. 2021;7(7):1156–65. doi: 10.1021/acscentsci.0c01537 34341769 PMC8265543

[59] Zhao J, Chen J, Li M, Chen M, Sun C. Multifaceted Functions of CH25H and 25HC to Modulate the Lipid Metabolism, Immune Responses, and Broadly Antiviral Activities. Viruses. 2020;12(7). doi: 10.3390/v12070727 32640529 PMC7411728

[60] Engeland K. Cell cycle regulation: p53-p21-RB signaling. Cell Death Differ. 2022;29(5):946–60. doi: 10.1038/s41418-022-00988-z 35361964 PMC9090780

[61] Scheurer L, Das Gupta RR, Saebisch A, Grampp T, Benke D, Zeilhofer HU, et al. Expression of immunoglobulin constant domain genes in neurons of the mouse central nervous system. Life Sci Alliance. 2021;4(11). doi: 10.26508/lsa.202101154 34433614 PMC8403770

[62] Leader AM, Grout JA, Maier BB, Nabet BY, Park MD, Tabachnikova A, et al. Single-cell analysis of human non-small cell lung cancer lesions refines tumor classification and patient stratification. Cancer Cell. 2021;39(12):1594-+. doi: 10.1016/j.ccell.2021.10.009 34767762 PMC8728963

[63] Dammeijer F, van Gulijk M, Mulder EE, Lukkes M, Klaase L, van den Bosch T, et al. The PD-1/PD-L1-Checkpoint Restrains T cell Immunity in Tumor-Draining Lymph Nodes. Cancer Cell. 2020;38(5):685–700 e8. doi: 10.1016/j.ccell.2020.09.001 33007259

[64] Gordon SR, Aute RLM, Dulken BW, Hutter G, George BM, Ccracken MNM, et al. PD-1 expression by tumour-associated macrophages inhibits phagocytosis and tumour immunity. Nature. 2017;545(7655):495-+. doi: 10.1038/nature22396 28514441 PMC5931375

[65] Chen Y, Jin H, Song Y, Huang T, Cao J, Tang Q, et al. Targeting tumor-associated macrophages: A potential treatment for solid tumors. J Cell Physiol. 2021;236(5):3445–65. doi: 10.1002/jcp.30139 33200401

[66] Cho JW, Hong MH, Ha SJ, Kim YJ, Cho BC, Lee I, et al. Genome-wide identification of differentially methylated promoters and enhancers associated with response to anti-PD-1 therapy in non-small cell lung cancer. Exp Mol Med. 2020;52(9):1550–63. doi: 10.1038/s12276-020-00493-8 32879421 PMC8080767

[67] Becht E, McInnes L, Healy J, Dutertre CA, Kwok IWH, Ng LG, et al. Dimensionality reduction for visualizing single-cell data using UMAP. Nat Biotechnol. 2018. doi: 10.1038/nbt.4314 30531897

[68] Luo Y, Chen Y, Jin H, Hou B, Li H, Li X, et al. The suppression of cervical cancer ferroptosis by macrophages: The attenuation of ALOX15 in cancer cells by macrophages-derived exosomes. Acta Pharm Sin B. 2023;13(6):2645–62. doi: 10.1016/j.apsb.2023.03.025 37425043 PMC10326300

[69] Stuart T, Butler A, Hoffman P, Hafemeister C, Papalexi E, Mauck WM, 3rd, et al. Comprehensive Integration of Single-Cell Data. Cell. 2019;177(7):1888–902 e21. doi: 10.1016/j.cell.2019.05.031 31178118 PMC6687398

[70] Butler A, Hoffman P, Smibert P, Papalexi E, Satija R. Integrating single-cell transcriptomic data across different conditions, technologies, and species. Nat Biotechnol. 2018;36(5):411–20. doi: 10.1038/nbt.4096 29608179 PMC6700744

[71] Jin S, Guerrero-Juarez CF, Zhang L, Chang I, Ramos R, Kuan CH, et al. Inference and analysis of cell-cell communication using CellChat. Nat Commun. 2021;12(1):1088. doi: 10.1038/s41467-021-21246-9 33597522 PMC7889871

[72] Colaprico A, Silva TC, Olsen C, Garofano L, Cava C, Garolini D, et al. TCGAbiolinks: an R/Bioconductor package for integrative analysis of TCGA data. Nucleic Acids Res. 2016;44(8):e71. doi: 10.1093/nar/gkv1507 26704973 PMC4856967

[73] Hugo W, Zaretsky JM, Sun L, Song C, Moreno BH, Hu-Lieskovan S, et al. Genomic and Transcriptomic Features of Response to Anti-PD-1 Therapy in Metastatic Melanoma. Cell. 2016;165(1):35–44. doi: 10.1016/j.cell.2016.02.065 26997480 PMC4808437

[74] He Y, Ramesh A, Gusev Y, Bhuvaneshwar K, Giaccone G. Molecular predictors of response to pembrolizumab in thymic carcinoma. Cell Rep Med. 2021;2(9):100392. doi: 10.1016/j.xcrm.2021.100392 34622229 PMC8484507

