# Supplementary figures and images for: Tumor-associated macrophage subtypes on cancer immunity along with prognostic analysis and SPP1-mediated interactions between tumor cells and macrophages
Source: PLoS Genet. 2024 Apr 22;20(4):e1011235. doi: 10.1371/journal.pgen.1011235 (PMC11034676; doi:10.1371/journal.pgen.1011235)

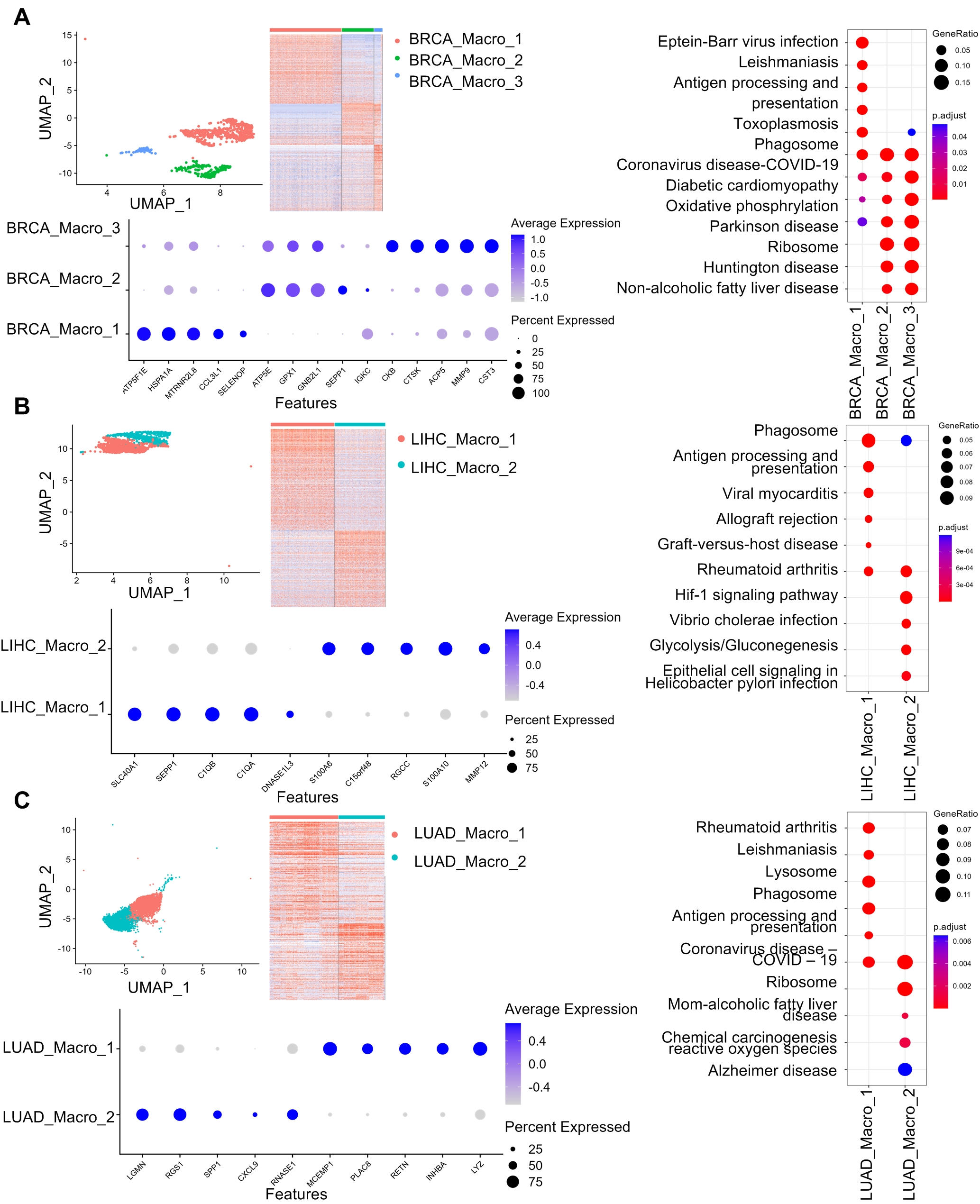

Supplement: S1 Fig — (A) UMAP distribution, expression of marker genes and gene enrichment analysis of macrophages in breast cancer. (B) Distribution of UMAP, expression of marker genes, and gene enrichment analysis of macrophages in hepatocellular carcinoma. (C) Distribution of UMAP, expression of marker genes, and gene enrichment analysis of macrophages in lung cancer. (TIF) [file pgen.1011235.s001.tif]

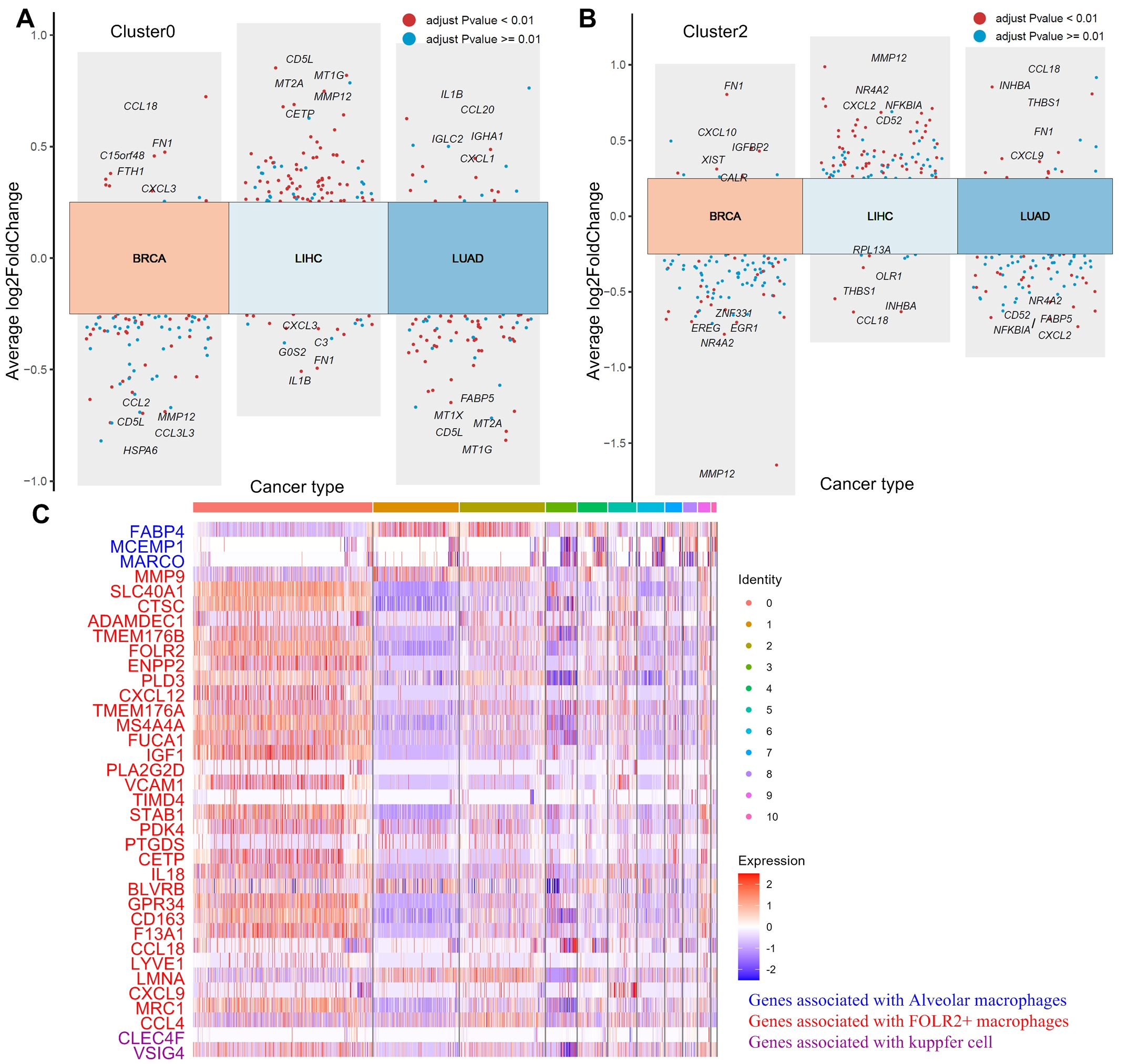

Supplement: S2 Fig — Differential gene volcano maps for different cancer types in Cluster0(A) and Cluster2 (B) (tissue-resident macrophages). (C) Expression of cancer-specific resident macrophage gene sets in different subpopulations. (TIF) [file pgen.1011235.s002.tif]

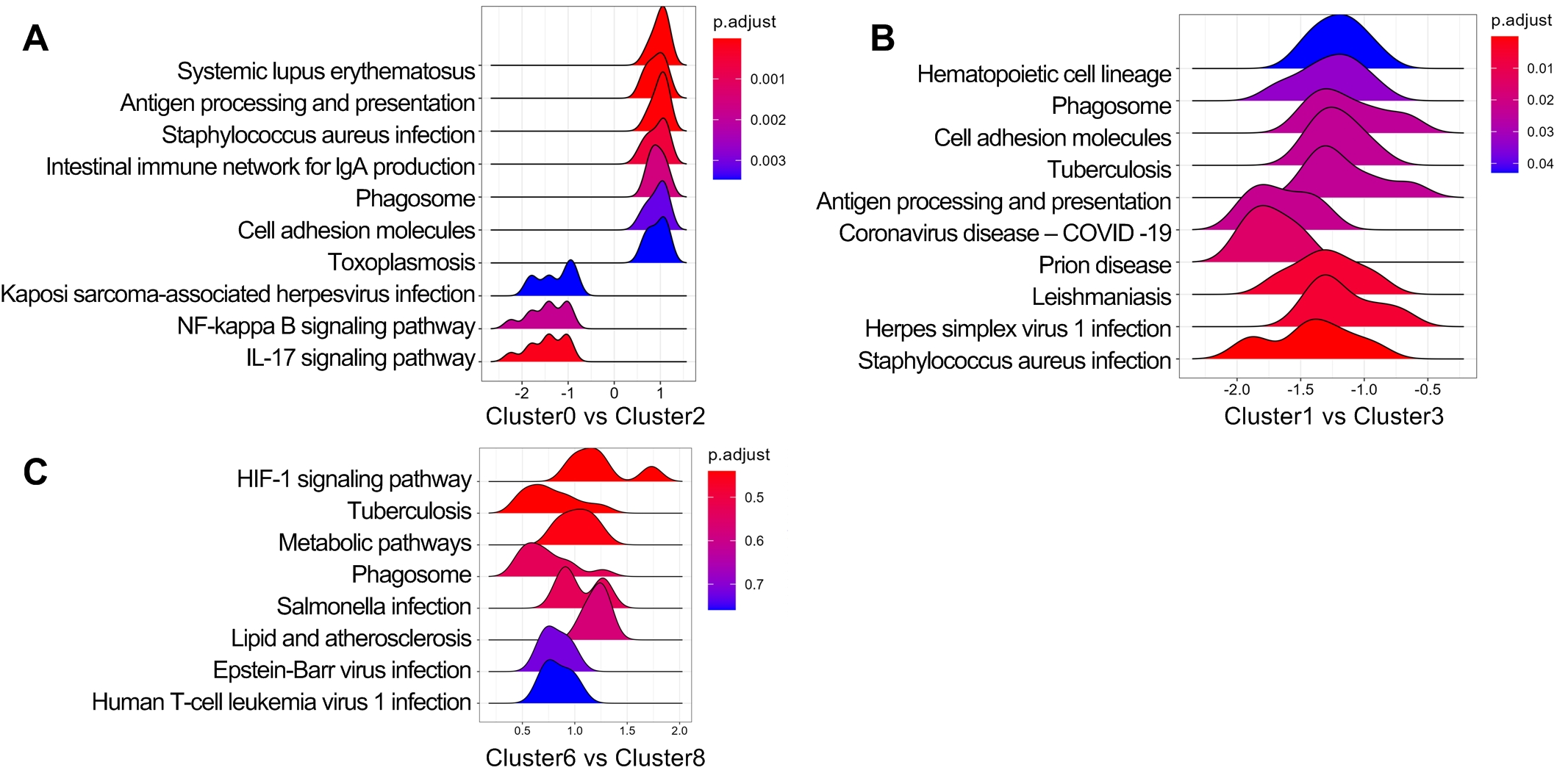

Supplement: S3 Fig — Functional differences between similar subgroups, (A) Cluster0 vs Cluster2, (B) Cluster1 vs Cluster3, (C) Cluster6 vs Cluster8. (TIF) [file pgen.1011235.s003.tif]

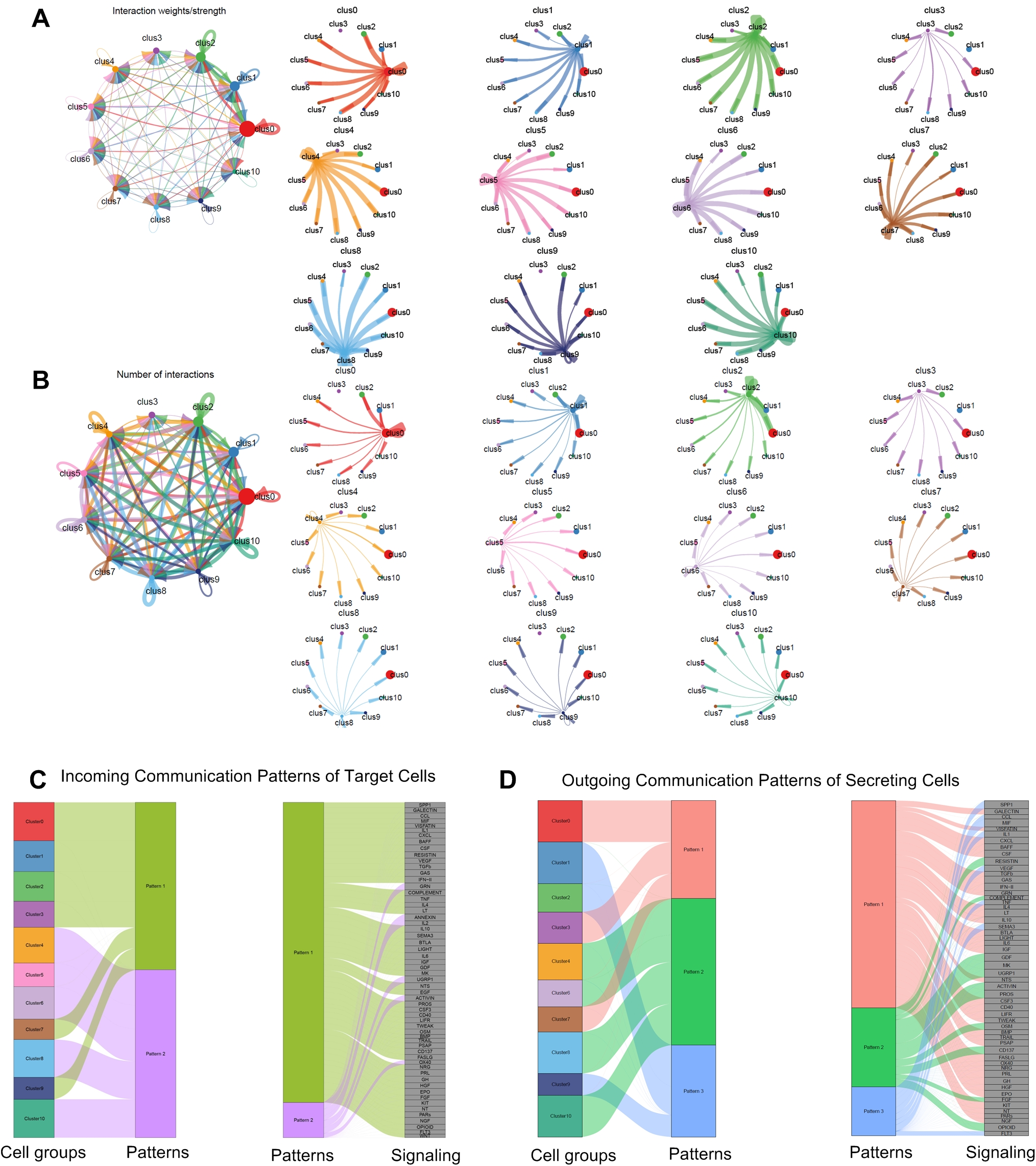

Supplement: S4 Fig — (A) The number of intercellular communications in different subpopulations, the greater the number, the thicker the linkage between different subgroups. (B) Intercellular communication strength of different subpopulations, the higher the intensity, the thicker the linkage between subgroups. (C) The communication patterns of intercellular inputs of different cell subpopulations. (D) The communication patterns of intercellular outgoings of different cell subpopulations. (TIF) [file pgen.1011235.s004.tif]

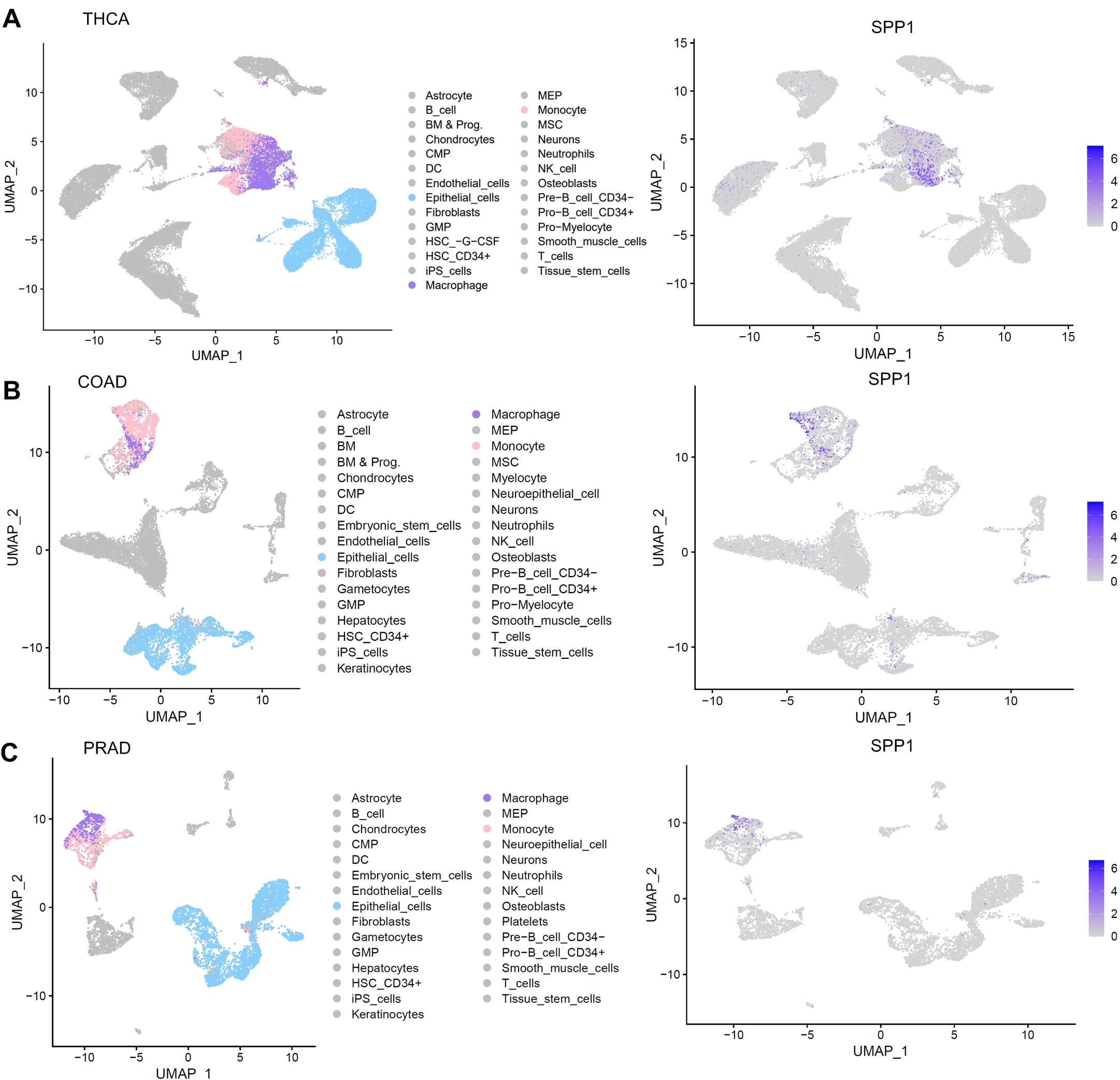

Supplement: S5 Fig — (A) UMAP distribution of cell types of THCA and UMAP showing SPP1 expression. (B) UMAP distribution of cell types of COAD and UMAP showing SPP1 expression. (C) UMAP distribution of cell types of PRAD and UMAP showing SPP1 expression (TIF) [file pgen.1011235.s005.tif]

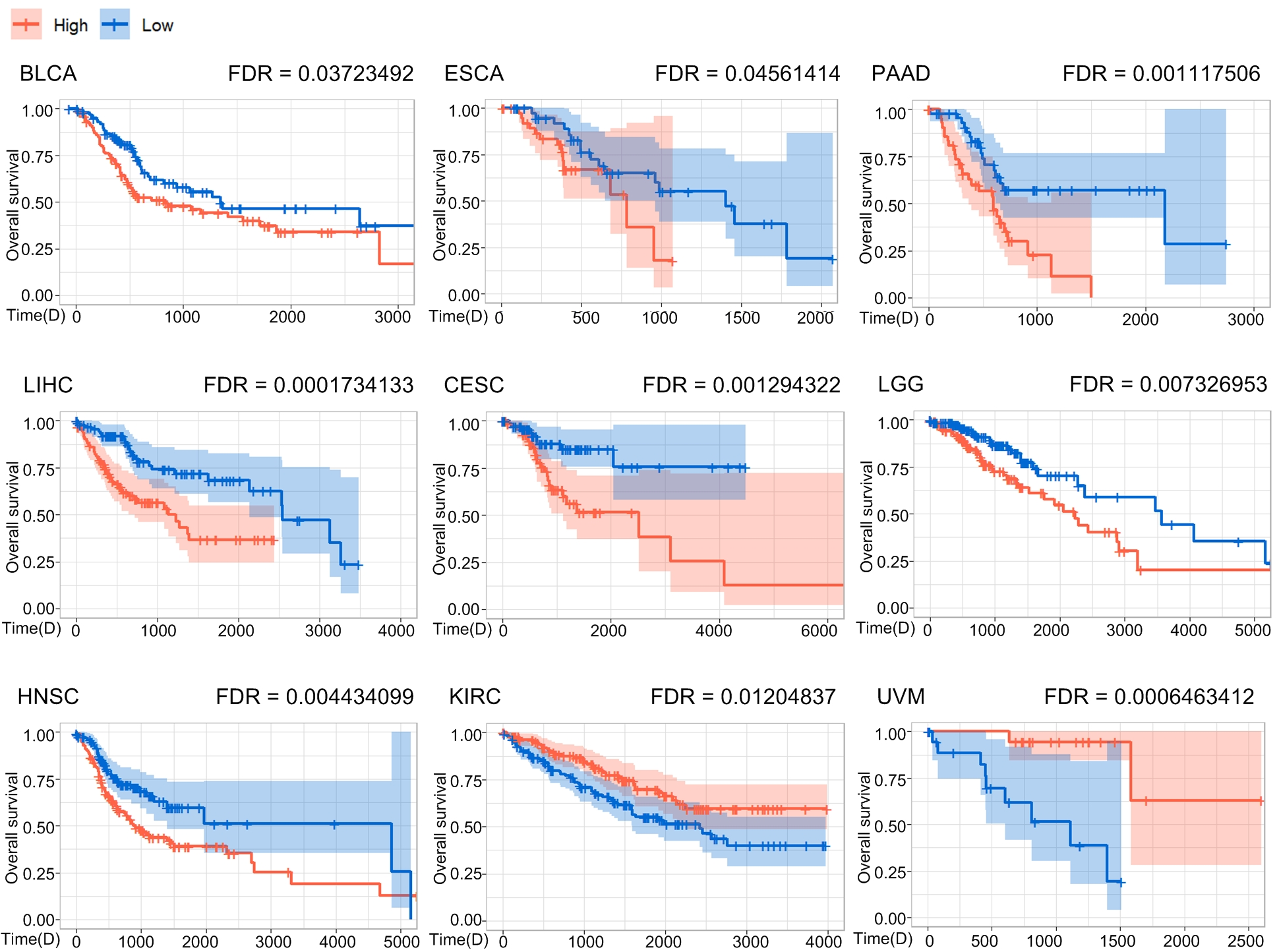

Supplement: S6 Fig — The light-colored background area is the confidence interval of the probability of survival at each time point calculated by the KM method, which is the 95% confidence interval, Time(D) = Time (Days). (TIF) [file pgen.1011235.s006.tif]

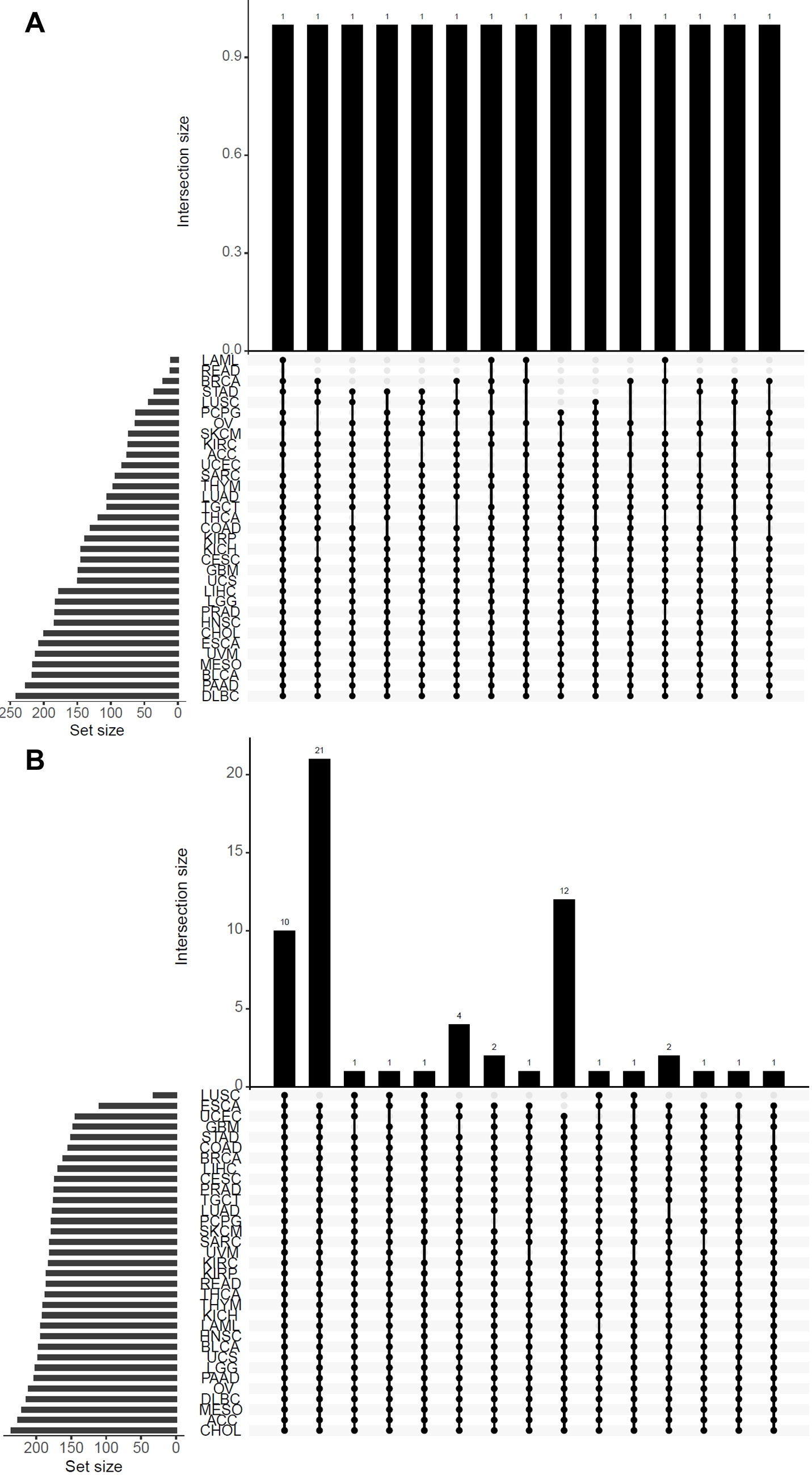

Supplement: S7 Fig — Intersection of enrichment pathways for different receptors in 33 cancers, CD44(A), ITGB1(B). (TIF) [file pgen.1011235.s007.tif]

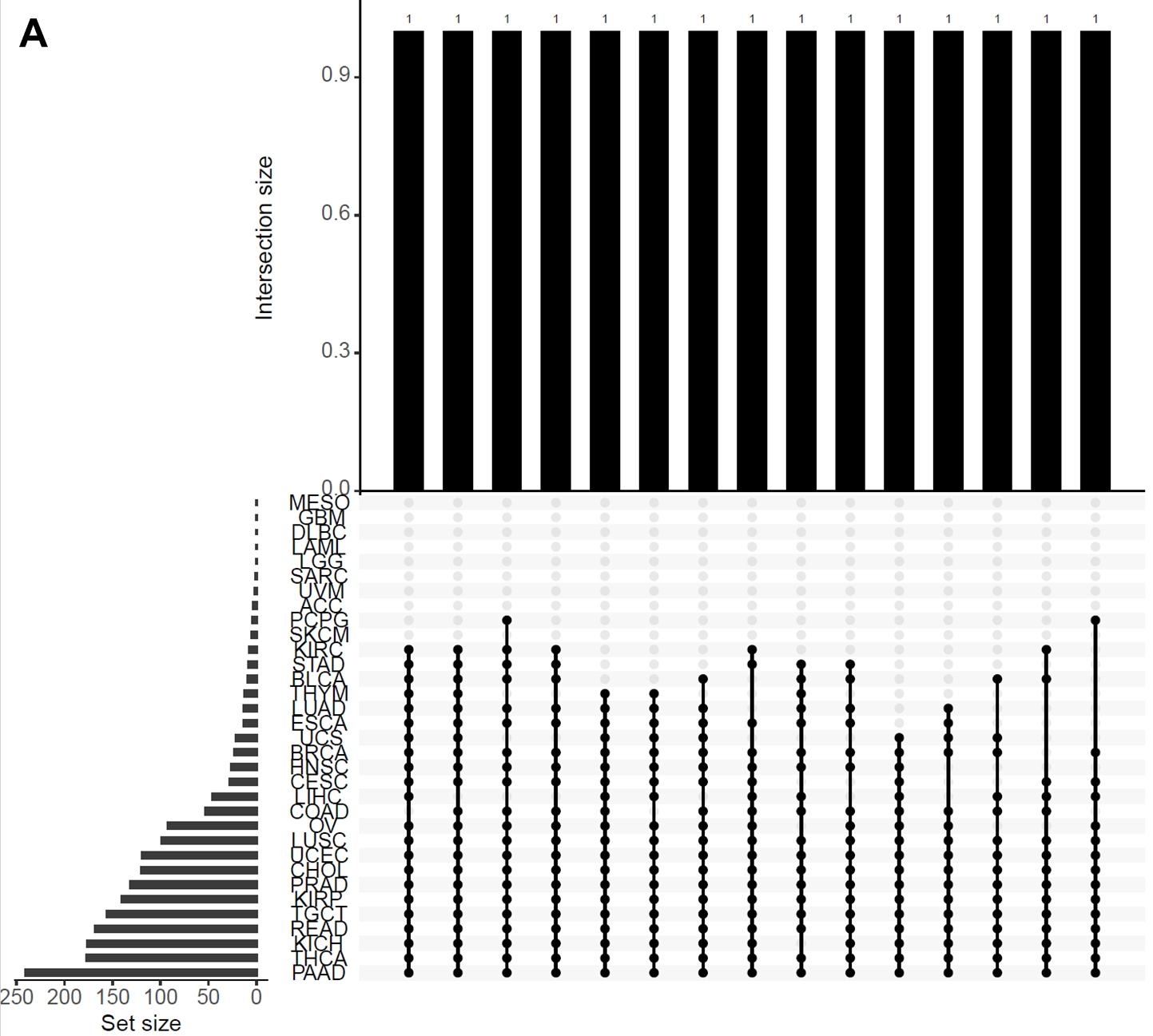

Supplement: S8 Fig — Intersection of ITGB6 receptor enrichment pathways in 33 cancers. (TIF) [file pgen.1011235.s008.tif]

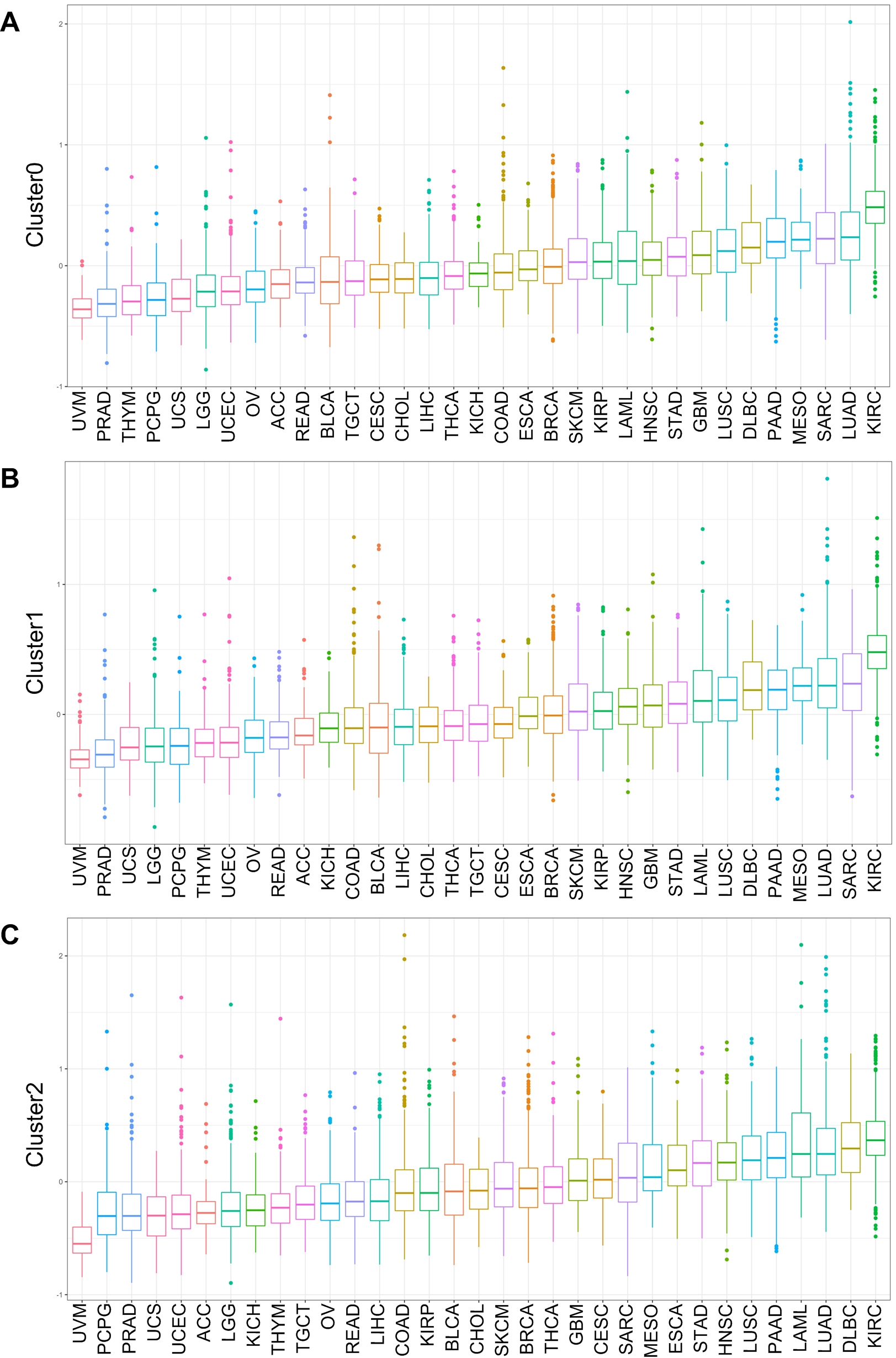

Supplement: S9 Fig — Expression of genes characteristic of macrophage subpopulations in 33 tumors in descending order, Cluster0 (A), Cluster1 (B), Cluster2 (C). (TIF) [file pgen.1011235.s009.tif]

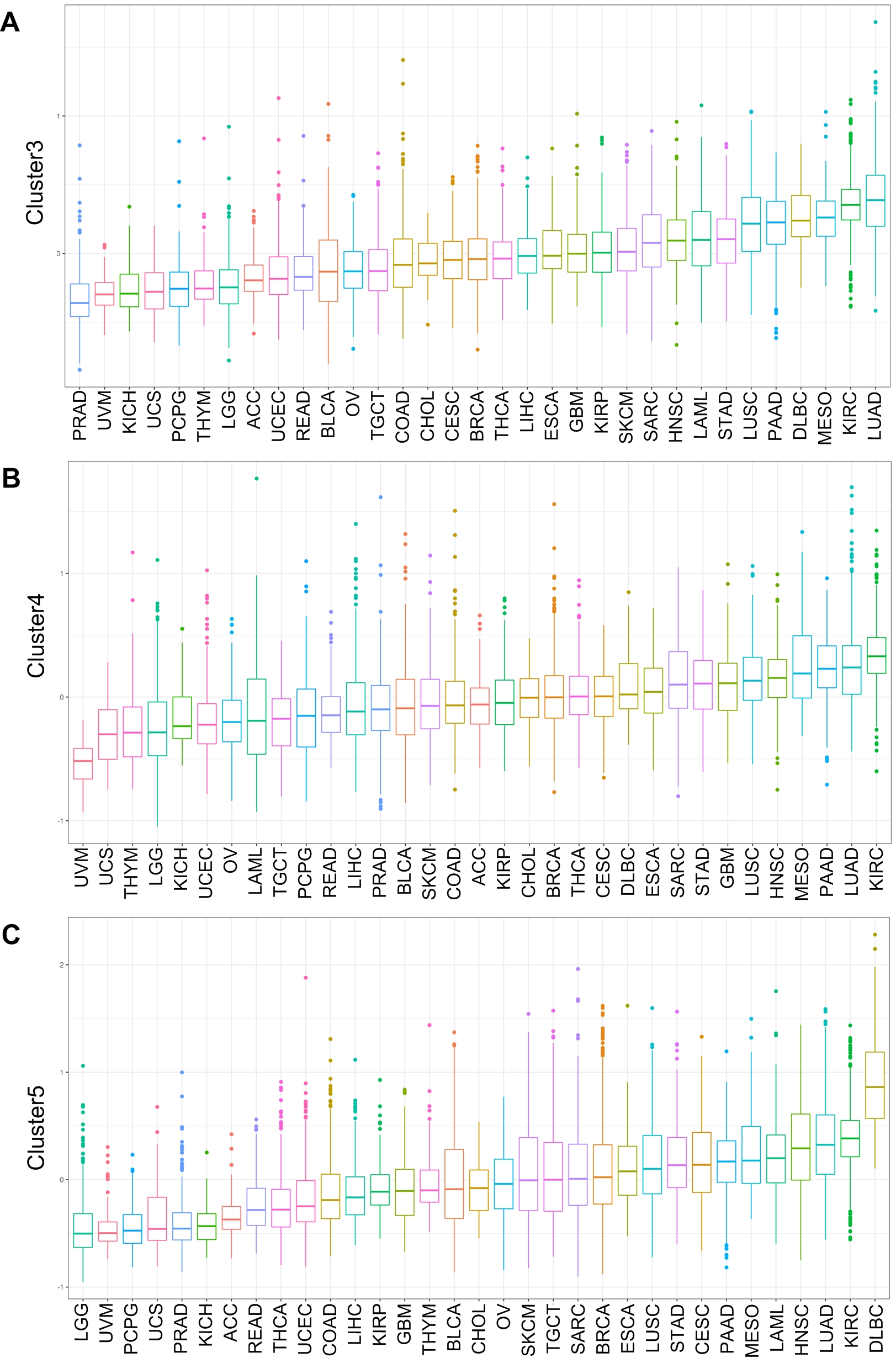

Supplement: S10 Fig — Expression of genes characteristic of macrophage subpopulations in 33 tumors in descending order, Cluster3 (A), Cluster4 (B), Cluster5 (C). (TIF) [file pgen.1011235.s010.tif]

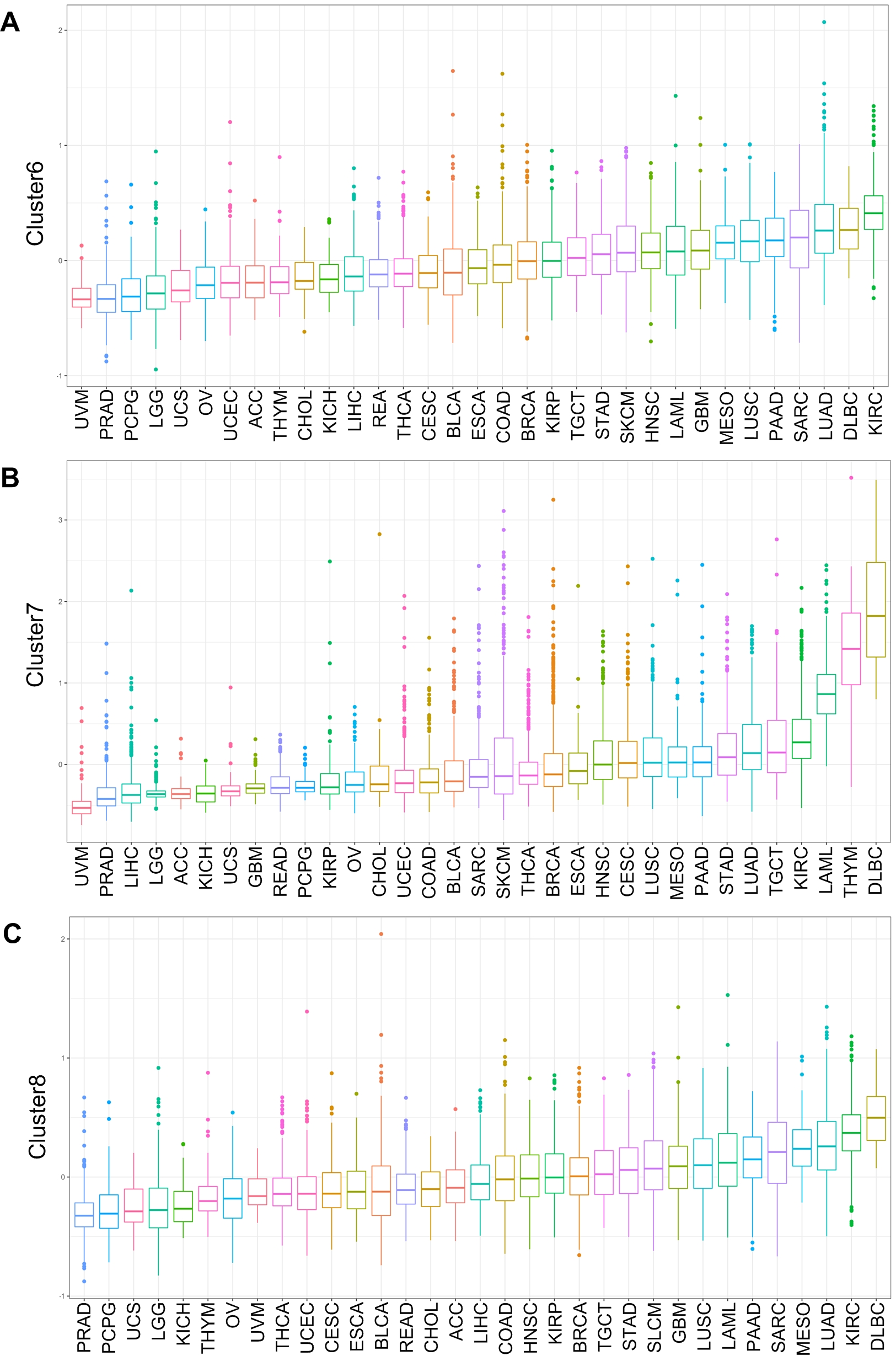

Supplement: S11 Fig — Expression of genes characteristic of macrophage subpopulations in 33 tumors in descending order, Cluster6 (A), Cluster7 (B), Cluster8 (C). (TIF) [file pgen.1011235.s011.tif]

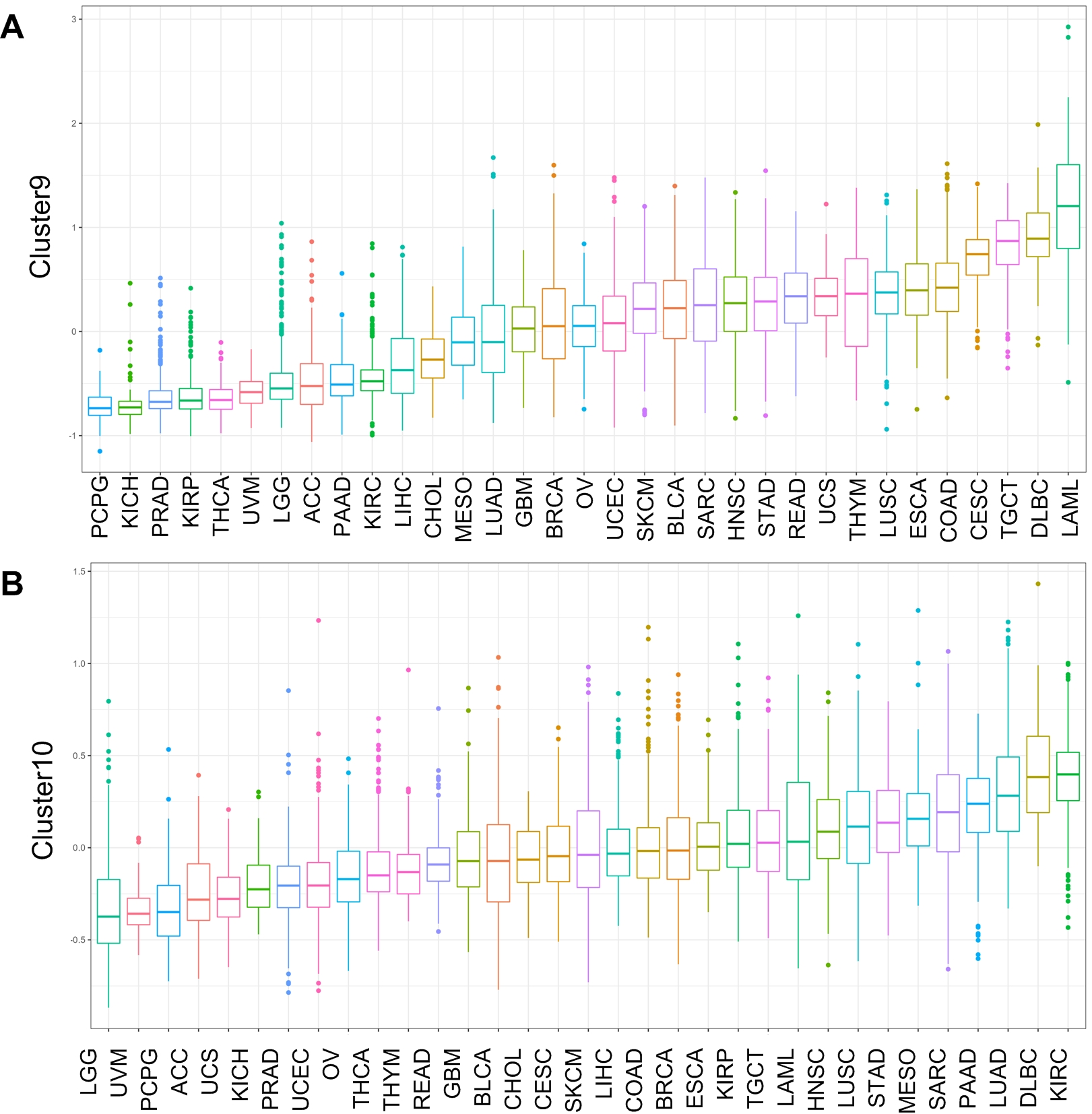

Supplement: S12 Fig — Expression of genes characteristic of macrophage subpopulations in 33 tumors in descending order, Cluster9 (A), Cluster10 (B). (TIF) [file pgen.1011235.s012.tif]

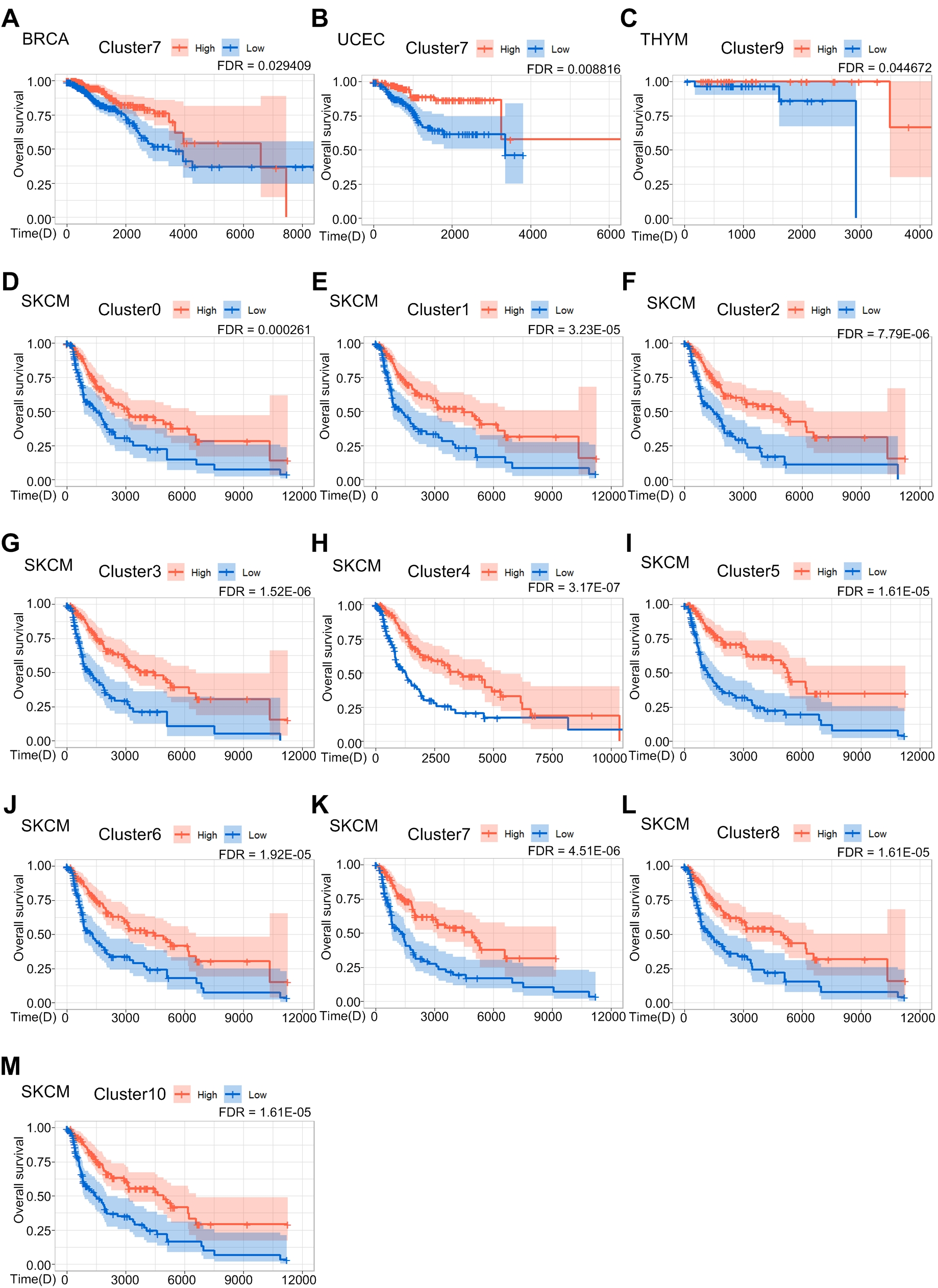

Supplement: S13 Fig — Kaplan-Meier curves depicting survival differences in high and low expression signature genes in multiple subpopulations for THYM (A), and SKCM (B-K), BRCA (L), UCEC (M). The light-colored background area is the confidence interval of the probability of survival at each time point calculated by the KM method, which is the 95% confidence interval, Time(D) = Time (Days). (TIF) [file pgen.1011235.s013.tif]

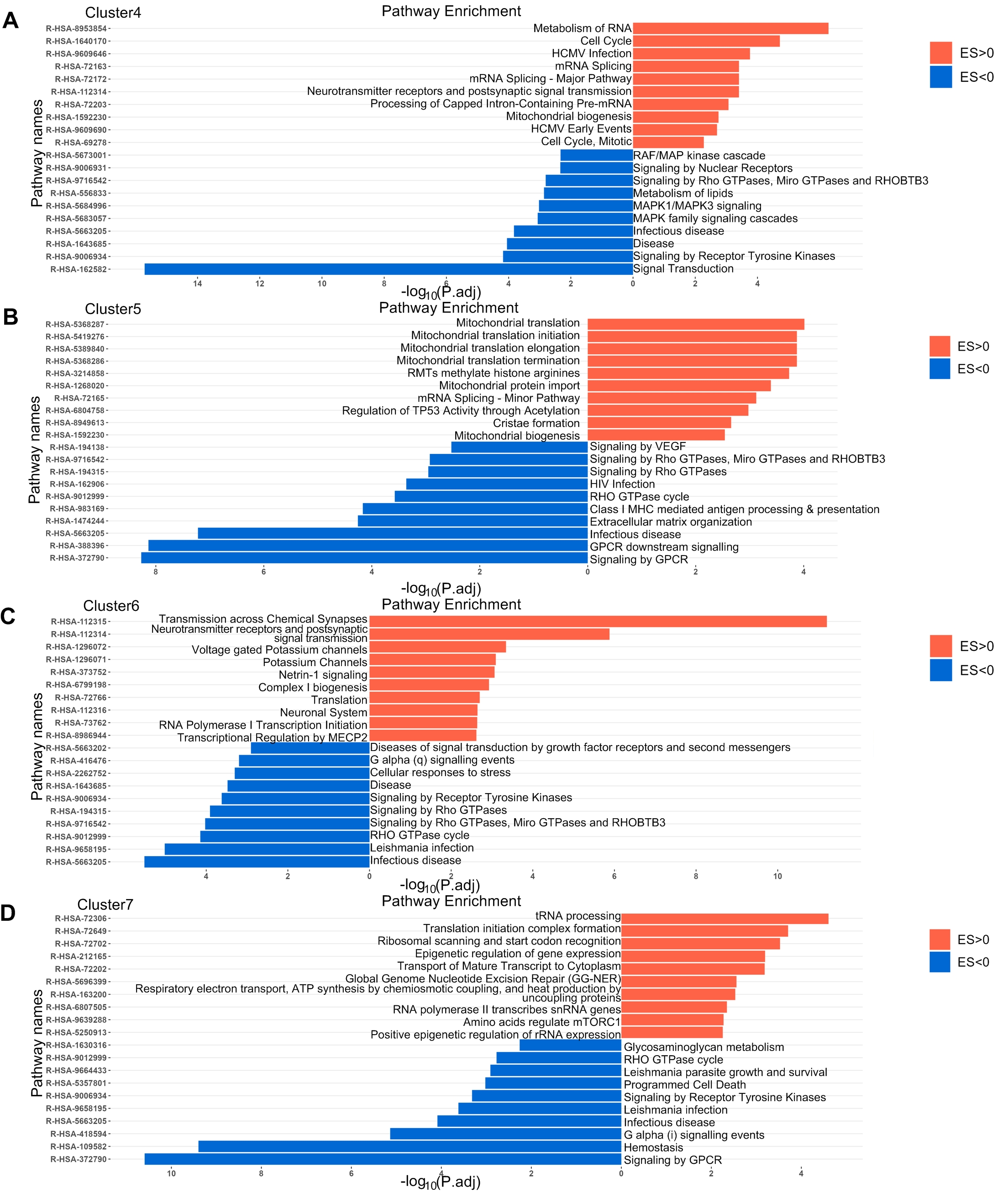

Supplement: S14 Fig — (A) Results of THYM, SKCM and LGG for Cluster4. (B) Results of PAAD, LGG, SKCM, THYM on Cluster5. (C) Results of SKCM, LGG, GBM in Cluster6. (D) Results of UCEC, BRCA, SKCM, LGG in Cluster7. (TIF) [file pgen.1011235.s014.tif]

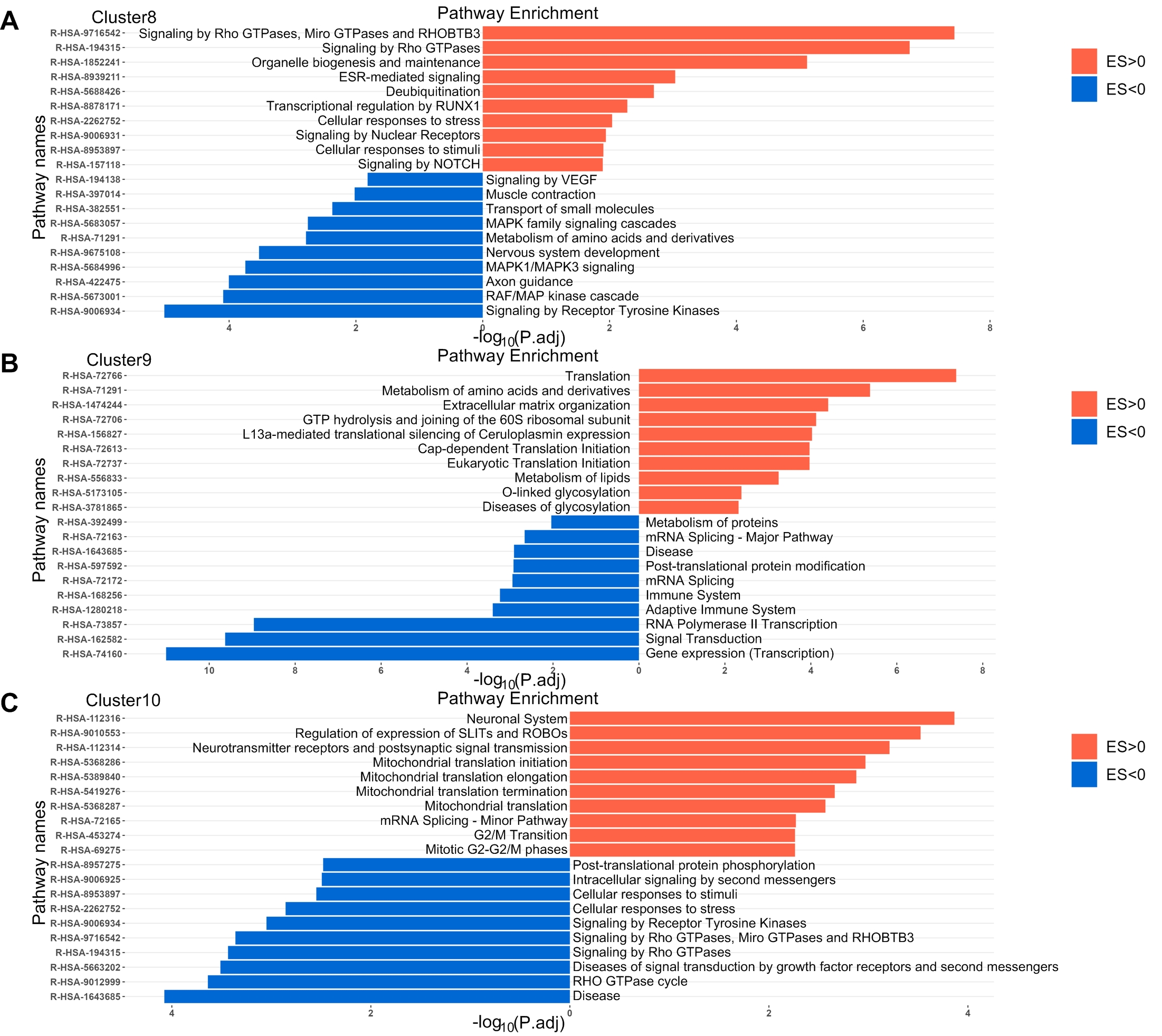

Supplement: S15 Fig — (A) Results of SKCM, LGG in Cluster8. (B) Results of LUAD, PAAD, KIRP, THYM, MESO, LGG, ACC, UCEC in Cluster 9. (C) Results for SKCM, LGG in Cluster10. (TIF) [file pgen.1011235.s015.tif]

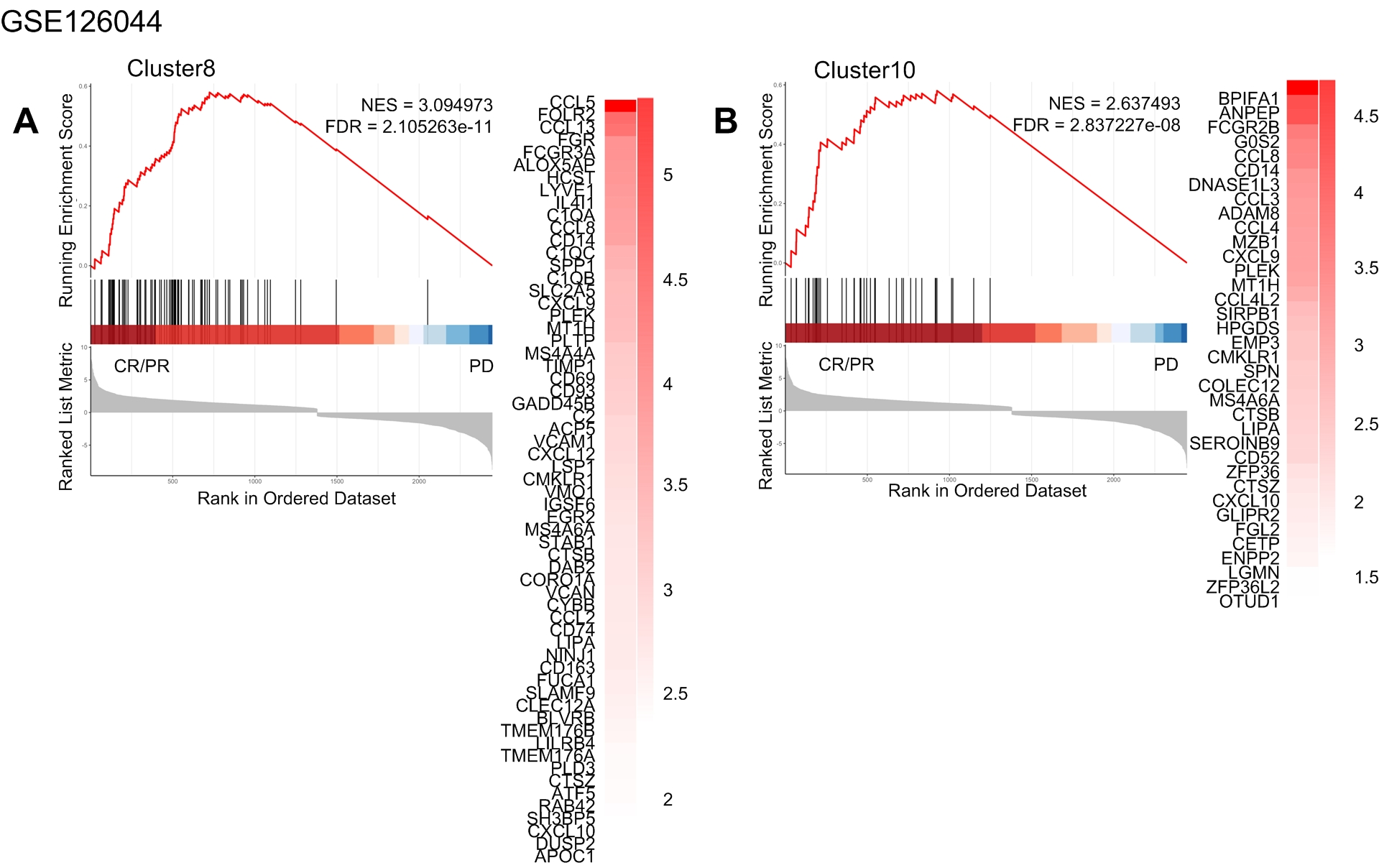

Supplement: S16 Fig — Gene set enrichment analysis of anti-PD 1 treatment for NSCLC of Cluster8 and Cluster10 showed that patients in complete remission (CR) and partial remission (PR) compared with those in patients with progressive disease (PD). (TIF) [file pgen.1011235.s016.tif]
